# Nucleic acids through condensation of nucleosides and phosphorous acid in the presence of sulfur

**DOI:** 10.3762/bjoc.12.67

**Published:** 2016-04-11

**Authors:** Tuomas Lönnberg

**Affiliations:** 1 Department of Chemistry, University of Turku, Vatselankatu 2, FIN-20014 Turku, Finland

**Keywords:** H-phosphonate, nucleic acid, polymerization, phosphite, sulfurization

## Abstract

Short phosphorothioate oligonucleotides have been prepared by refluxing an equimolar mixture of thymidine and triethylammonium phosphite in toluene in the presence of elemental sulfur. Desulfurization and subsequent digestion of the products by P1 nuclease revealed that nearly 80% of the internucleosidic linkages thus formed were of the canonical 3´,5´-type.

## Introduction

Arguably the most crucial step in the origin of life was the prebiotic formation of information carrying polymers. For the polymers playing this role in contemporary biochemistry, i.e., DNA and RNA, such a process appears difficult owing to the low reactivity and solubility of phosphate. Indeed, all of the enzyme-free nucleic acid polymerizations described so far have utilized activated starting materials, such as cyclic phosphates [1–2] or phosphoroimidazolides [3–4].

The trivalent phosphorus atom of phosphorous acid is much more susceptible to a nucleophilic attack than the pentavalent phosphorus atom of phosphoric acid [5]. Furthermore, phosphite salts are up to 1000-fold more soluble in water than phosphate salts [6]. For these reasons, compounds of reduced phosphorus (i.e., phosphorus at oxidation state lower than +5) were first proposed to have played a role in prebiotic phosphorylation reactions as early as 1955 [7]. Since then, both terrestrial [8] and extraterrestrial [9] sources of reduced phosphorus have been identified. Recent studies suggest the presence of significant amounts of phosphite in the Archean ocean, lending support to the idea of prebiotic phosphite chemistry [10–11].

Monoesters of phosphorous acid are hydrolytically stable compounds that are readily formed upon concentration of aqueous solutions of alcohols and phosphite salts [12]. The monoesters may react further to H-phosphonate diesters but this is an equilibrium process that under aqueous conditions favors the starting materials [13]. Oxidation of the H-phosphonate diester products, however, converts them to the stable phosphodiester counterparts. It is interesting to note that this reaction is faster for H-phosphonate diesters than for the respective monoesters or phosphorous acid itself [12], providing the driving force for polymerization. Proposed oxidants for prebiotic phosphite chemistry include H_2_O_2_ and Fe(III).

The present paper describes the polymerization of thymidine and triethylammonium phosphite, with elemental sulfur acting as the oxidant. Up to pentameric oligonucleotides with internucleosidic phosphorothioate linkages of predominantly 3´,5´-regiochemistry were formed by this method. Elemental sulfur may have been abundant on primitive Earth but its availability is limited by its poor solubility. In the present study this problem was addressed by carrying out the model reaction in toluene. On primitive Earth, solubilization by micelles [14–15] or a prebiotic oil slick [16] appears a more plausible scenario [17].

## Results and Discussion

### Preparation of phosphorothioate oligonucleotides

**Figure 1:**
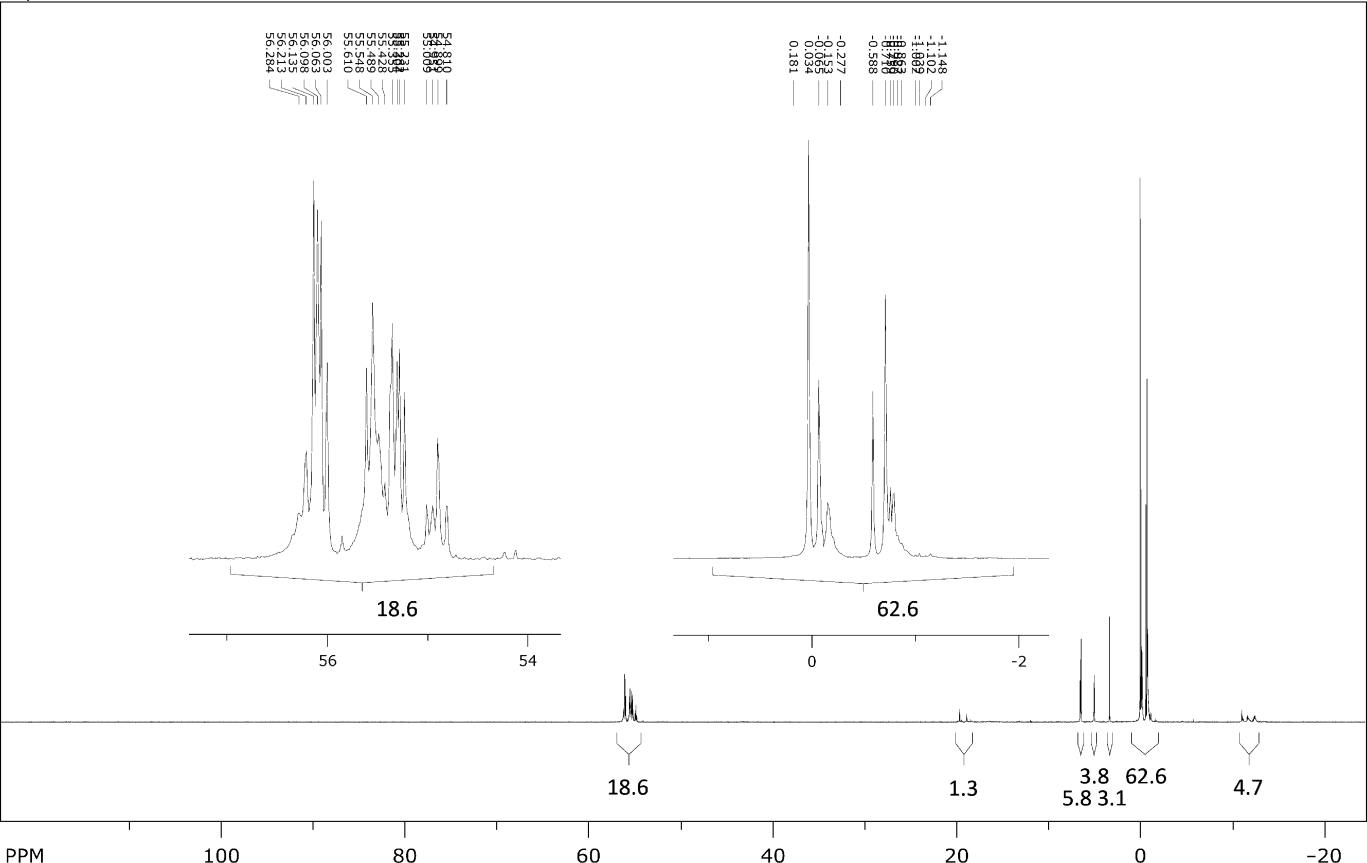
^31^P NMR spectrum (162 MHz, D_2_O) of the crude product mixture after refluxing equimolar amounts of thymidine and triethylammonium phosphite and 4 equiv of S_8_ in toluene for 90 h.

Equimolar amounts of thymidine and aq triethylammonium phosphite and a fourfold excess of S_8_ were suspended in toluene. The mixture was refluxed at 130 °C in a Dean–Stark apparatus for 90 h, after which it was evaporated to dryness. The brown glassy residue was suspended in water and the resulting mixture filtered. A sample of the filtrate was evaporated to dryness and the residue analyzed by ^31^P NMR (Figure 1). According to the ^31^P NMR spectrum, approximately 18% of the phosphite starting material had been converted to diverse phosphorothioate products.

**Figure 2:**
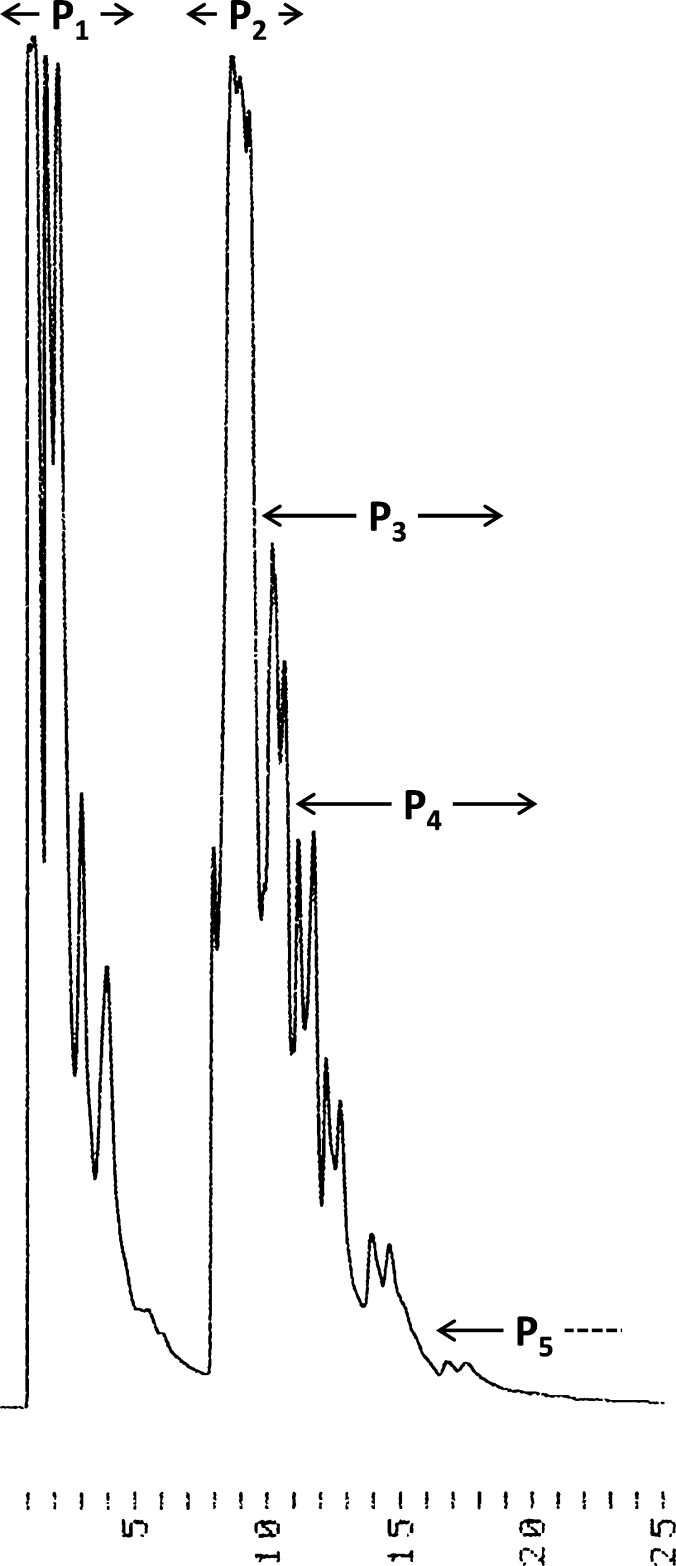
IE HPLC trace of the crude product mixture after refluxing equimolar amounts of thymidine and triethylammonium phosphite and 4 equiv of S_8_ in toluene for 90 h; Dionex DNASwift^TM^ column (150 × 5 mm, monolithic); flow rate = 1.5 mL min^−1^; linear gradient (0 to 50% over 25 min) of 330 mM NaClO_4_ in 20 mM TRIS buffer (pH 7.0).

The product mixture was fractioned first by ion-exchange (IE) and then by reversed-phase (RP) HPLC (Figure 2, RP chromatograms presented in Supporting Information File 1). Electrospray ionization mass spectrometric (ESIMS) analysis of the collected fractions suggested the main products to be short oligonucleotides with internucleosidic phosphorothioate linkages. As could be expected, retention times in the IE HPLC correlated with the number of the negatively charged phosphorus-containing groups (Figure 2). While the shortest oligonucleotides (dimers and trimers) predominated in the mixture, up to pentameric products could be identified.

**Figure 3:**
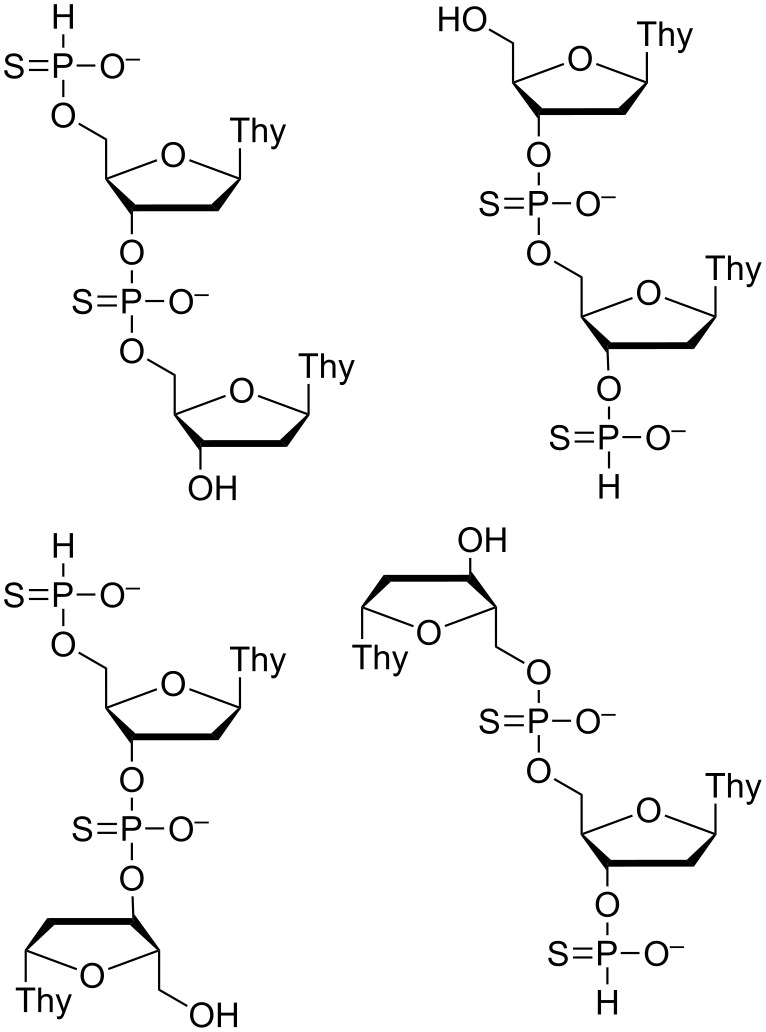
Possible structures of the most abundant product.

All of the expected phosphorothioate oligonucleotides were accompanied by slower eluting (in IE HPLC) products exhibiting molecular weights 80 or 160 Da higher than the parent oligonucleotide. Similar derivatives of monomeric thymidine were also detected in the fastest eluting fractions. The 80 Da difference in molecular weight could arise from capping of a free hydroxy function by a phosphate or an H-phosphonothioate group and distinguishing between these two alternatives on the basis of MS alone is challenging. To verify the structure of the capping groups, a sample of the most abundant product, comprising two thymidines and two phosphorus atoms, was analyzed by ^31^P NMR. Phosphorothioate and H-phosphonothioate signals accounted for approximately 90% of the overall intensity (data presented in Supporting Information File 1), suggesting the product to be a bis(thymidinyl)phosphorothioate diester with one of the free hydroxy functions capped by an H-phosphonothioate group (Figure 3). It seems likely that also the other capped oligonucleotides had H-phosphonothioate, rather than phosphate termini. Evidently sulfurization and esterification of H-phosphonate monoesters compete under the experimental conditions.

### Determination of the regiochemistry of the phosphorothioate linkages

**Figure 4:**
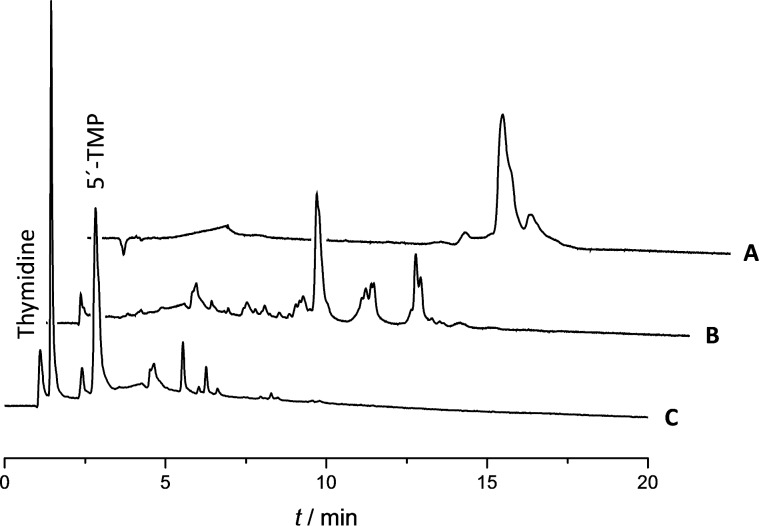
IE HPLC traces of (A) a mixture of tetrameric products, (B) the product mixture after desulfurization by iodine and (C) the product mixture after P1 nuclease digestion of the desulfurized material; Dionex DNASwift^TM^ column (150 × 5 mm, monolithic); flow rate = 1.5 mL min^−1^; linear gradient (2 to 35% over 20 min) of 330 mM NaClO_4_ in 20 mM TRIS buffer (pH 7.0). The tall peak eluting at 1.5 min in chromatogram C corresponds to either the enzyme itself or an impurity in the commercial preparation.

**Scheme 1:**
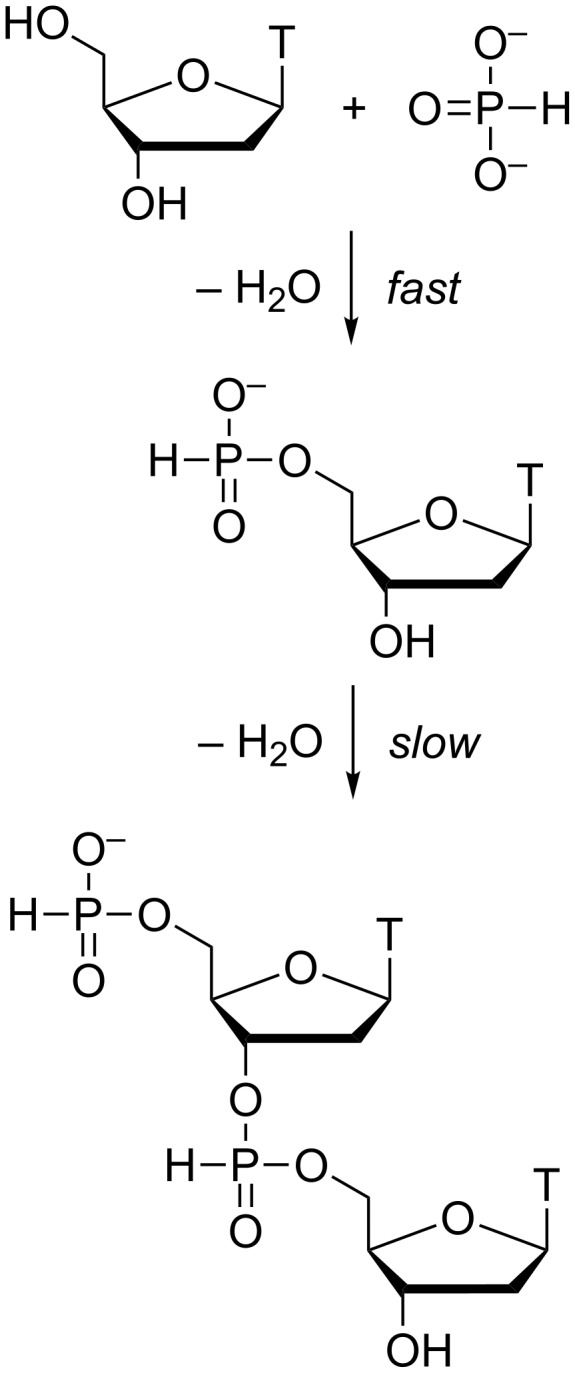
Phosphitylation and subsequent dimerization of thymidine.

To establish the regiochemistry of the phosphorothioate linkages formed, a purified sample consisting of a mixture of tetrameric products (Figure 4A) was first desulfurized by treatment with iodine in aq pyridine (Figure 4B). According to a previous report, phosphodiester linkages are stable under these conditions [18]. The resulting phosphate-linked oligonucleotides were then subjected to digestion by P1 nuclease (Figure 4C). Thymidine and thymidine-5´-monophosphate accounted for approximately 78% of the final product mixture. As cleavage by P1 nuclease is limited to 3´,5´-phosphodiester linkages of single-stranded DNA or RNA, the results of the digestion experiment indicate that nearly 80% of the internucleosidic linkages formed upon condensation of thymidine and triethylammonium phosphite in the presence of sulfur had the natural 3´,5´-regiochemistry. The most likely explanation for this regioselectivity is the faster phosphitylation of the primary 5´-hydroxy function [12] – most of the thymidine starting material is first converted to thymidine-5´-H-phosphonate that subsequently polymerizes (Scheme 1).

## Conclusion

Under dehydrating conditions and in the presence of sulfur, thymidine and triethylammonium phosphite condense into oligonucleotides with internucleosidic phosphorothioate linkages. Nearly 80% of these linkages have the natural 3´,5´-regiochemistry. Together with the recent evidence of phosphite in the Archean ocean, these results lend support to the hypothesis that phosphorous acid and its salts may have played a key role in the prebiotic synthesis of nucleic acids.

## Supporting Information

File 1 Experimental methods, HPLC chromatograms and mass spectra of the oligomerization products.
