# Supplementary material for: Nucleic acids through condensation of nucleosides and phosphorous acid in the presence of sulfur
Source: Beilstein J Org Chem. 2016 Apr 11;12:670–3. doi: 10.3762/bjoc.12.67 (PMC4902050; doi:10.3762/bjoc.12.67)
Supplement: File 1 — Experimental methods, HPLC chromatograms and mass spectra of the oligomerization products. [file Beilstein_J_Org_Chem-12-670-s001.pdf]

Supporting Information

for

Nucleic acids through condensation of nucleosides and phosphorous acid in the presence of sulfur

Tuomas Lönnberg*[1]

Address: [1]Department of Chemistry, University of Turku, Vatselankatu 2, FIN-20014 Turku, Finland

Email: Tuomas Lönnberg - tuanlo@utu.fi

*Corresponding author

Experimental methods, HPLC chromatograms and mass spectra of the oligomerization products

Contents

General experimental methods	S3
Oligomerization procedure	S3
**Figure S1** IE HPLC trace of the crude product mixture.	S3
**Figure S2** RP HPLC trace of fraction A of the IE HPLC fractioning of the crude product mixture.	S4
**Figure S3** RP HPLC trace of fraction B of the IE HPLC fractioning of the crude product mixture.	S4
**Figure S4** RP HPLC trace of fraction C of the IE HPLC fractioning of the crude product mixture.	S5
**Figure S5** RP HPLC trace of fraction D of the IE HPLC fractioning of the crude product mixture.	S5
**Figure S6** RP HPLC trace of fraction E of the IE HPLC fractioning of the crude product mixture.	S6
**Figure S7** RP HPLC trace of fraction F of the IE HPLC fractioning of the crude product mixture.	S6
**Figure S8** RP HPLC trace of fraction G of the IE HPLC fractioning of the crude product mixture.	S7
**Figure S9** RP HPLC trace of fraction H of the IE HPLC fractioning of the crude product mixture.	S7
**Figure S10** Electrospray ionization mass spectrum of fraction A1.	S8
**Figure S11** Electrospray ionization mass spectrum of fraction A2.	S8
**Figure S12** Electrospray ionization mass spectrum of fraction A3.	S9
**Figure S13** Electrospray ionization mass spectrum of fraction B1.	S9
**Figure S14** Electrospray ionization mass spectrum of fraction B2.	S10
**Figure S15** Electrospray ionization mass spectrum of fraction B3.	S10
**Figure S16** Electrospray ionization mass spectrum of fraction B4.	S11
**Figure S17** Electrospray ionization mass spectrum of fraction B5.	S11
**Figure S18** Electrospray ionization mass spectrum of fraction C1.	S12
**Figure S19** Electrospray ionization mass spectrum of fraction C2.	S12
**Figure S20** Electrospray ionization mass spectrum of fraction C3.	S13
**Figure S21** Electrospray ionization mass spectrum of fraction C4.	S13
**Figure S22** Electrospray ionization mass spectrum of fraction C5.	S14
**Figure S23** Electrospray ionization mass spectrum of fraction D1.	S14
**Figure S24** Electrospray ionization mass spectrum of fraction D2.	S15
**Figure S25** Electrospray ionization mass spectrum of fraction D3.	S15
**Figure S26** Electrospray ionization mass spectrum of fraction E1.	S16
**Figure S27** Electrospray ionization mass spectrum of fraction E2.	S16
**Figure S28** Electrospray ionization mass spectrum of fraction E3.	S17
**Figure S29** Electrospray ionization mass spectrum of fraction E4.	S17
**Figure S30** Electrospray ionization mass spectrum of fraction E5.	S18
**Figure S31** Electrospray ionization mass spectrum of fraction F2.	S18
**Figure S32** Electrospray ionization mass spectrum of fraction F3.	S19
**Figure S33** Electrospray ionization mass spectrum of fraction F4.	S19
**Figure S34** Electrospray ionization mass spectrum of fraction F5.	S20
**Figure S35** Electrospray ionization mass spectrum of fraction G2.	S20
**Figure S36** Electrospray ionization mass spectrum of fraction G3.	S21
**Figure S37** Electrospray ionization mass spectrum of fraction G4.	S21
**Figure S38** Electrospray ionization mass spectrum of fraction G5.	S22
**Figure S39** Electrospray ionization mass spectrum of fraction H2.	S22
Desulfurization and enzymatic digestion of the phosphorothioate oligonucleotide products.	S23

General experimental methods

Mass spectra were recorded on a Bruker Daltonics micrOTOF-Q ESI-MS spectrometer and NMR spectra on a Bruker Avance 400 MHz NMR spectrometer. The ^{31}P chemical shifts were referenced to external orthophosphoric acid and are given in ppm. For the preparation of the RP HPLC elution buffers, freshly distilled triethylamine was used. The other chemicals were commercial products that were used as received.

Oligomerization procedure

Thymidine (86.1 mg, 0.355 mmol), 0.34 M aq triethylammonium phosphite (1.0 mL, 0.34 mmol, pH 7.0) and S_8 (0.39 g, 1.52 mmol) were suspended in toluene (10 mL). The mixture was refluxed in a Dean-Stark apparatus at 125 °C for 90 h, after which it was evaporated to dryness. The residue was suspended in water and passed through a 0.2 µm PTFE membrane filter. The filtrate was first fractioned by anion-exchange HPLC on a Dionex DNASwift™ column (150 × 5 mm, monolithic) eluting with a linear gradient (0 to 50% over 25 min) of 330 mM $NaClO_4$ in 20 mM TRIS buffer (pH 7.0). The flow rate was 1.5 mL min^{-1} and the detection wavelength 260 nm. Eight fractions (labeled A–H in Figure S1) were collected and further fractioned by reversed-phase HPLC on a Thermo Scientific ODS Hypersil column (250 × 4.6 mm, 5 µm) eluting with a linear gradient (10 to 40% over 25 min) of MeCN in 100 mM aq triethylammonium acetate (pH 7.0). The flow rate was 1.0 mL min^{-1} and the detection wavelength 260 nm. The collected fractions (Figures S2–S9) were lyophilized and analyzed by ESIMS (Figures S10–S39, spectra are only included for the fractions in which phosphitylated products could be unambiguously identified).

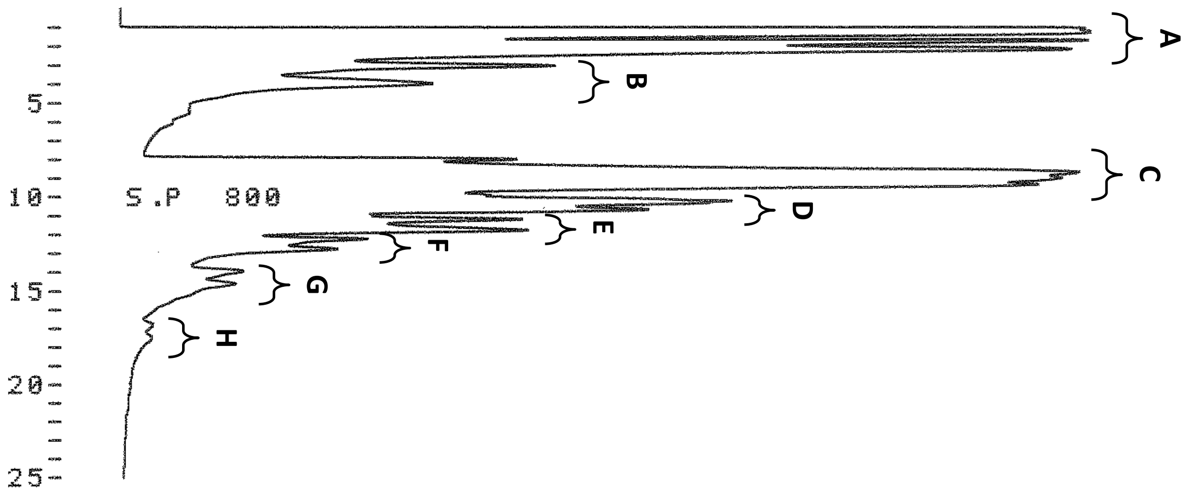

Figure S1: IE HPLC trace of the crude product mixture after refluxing equimolar amounts of thymidine and triethylammonium phosphite and 4 equiv of S_8 in toluene for 90 h; Dionex DNASwift™ column (150 × 5 mm, monolithic); flow rate = 1.5 mL min^{-1}; linear gradient (0 to 50% over 25 min) of 330 mM $NaClO_4$ in 20 mM TRIS buffer (pH 7.0). The collected fractions are labeled A–H.

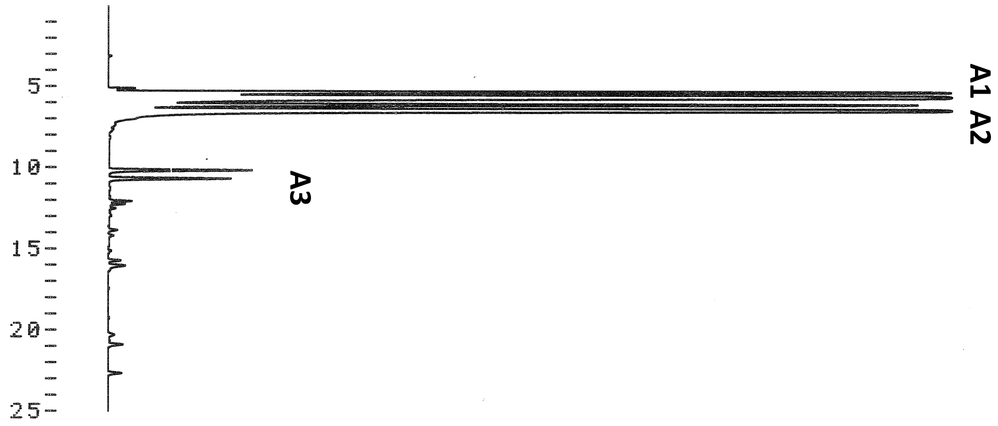

Figure S2: RP HPLC trace of fraction A of the IE HPLC fractioning of the crude product mixture; Thermo Scientific ODS Hypersil column (250 × 4.6 mm, 5 μm); flow rate = 1.0 mL min^{-1}; linear gradient (10 to 40% over 25 min) of MeCN in 100 mM aq triethylammonium acetate (pH 7.0). The collected fractions are labeled A1–A3.

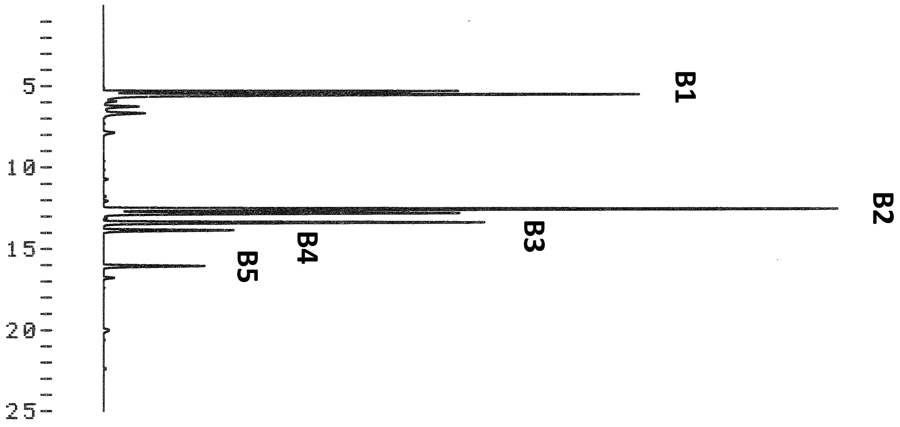

Figure S3: RP HPLC trace of fraction B of the IE HPLC fractioning of the crude product mixture; Thermo Scientific ODS Hypersil column (250 × 4.6 mm, 5 μm); flow rate = 1.0 mL min^{-1}; linear gradient (10 to 40% over 25 min) of MeCN in 100 mM aq triethylammonium acetate (pH 7.0). The collected fractions are labeled B1–B5.

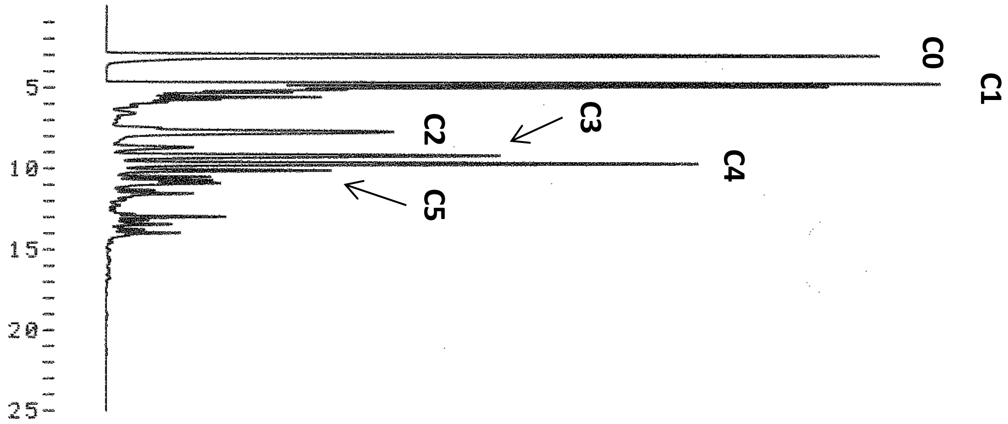

Figure S4: RP HPLC trace of fraction C of the IE HPLC fractioning of the crude product mixture; Thermo Scientific ODS Hypersil column (250 × 4.6 mm, 5 µm); flow rate = 1.0 mL min^{-1}; linear gradient (10 to 40% over 25 min) of MeCN in 100 mM aq triethylammonium acetate (pH 7.0). The collected fractions are labeled C1–C5.

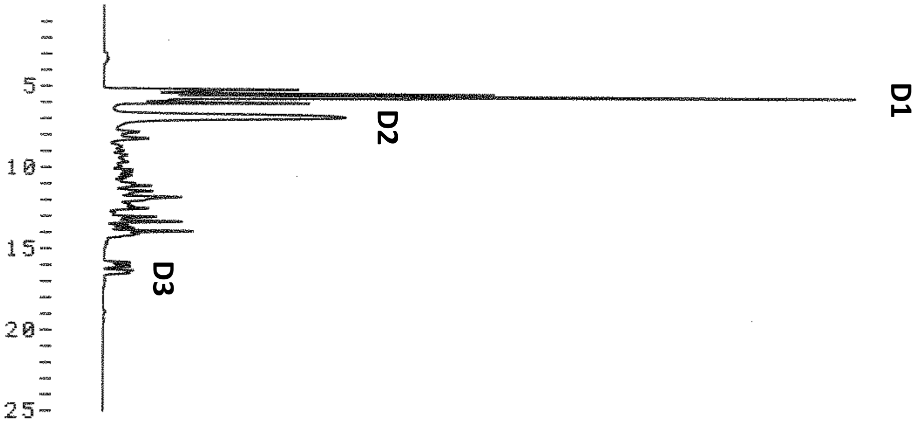

Figure S5: RP HPLC trace of fraction D of the IE HPLC fractioning of the crude product mixture; Thermo Scientific ODS Hypersil column (250 × 4.6 mm, 5 µm); flow rate = 1.0 mL min^{-1}; linear gradient (10 to 40% over 25 min) of MeCN in 100 mM aq triethylammonium acetate (pH 7.0). The collected fractions are labeled D1–D3.

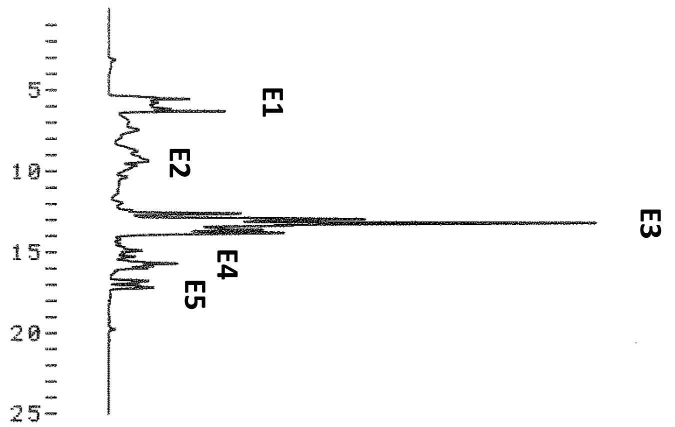

Figure S6: RP HPLC trace of fraction E of the IE HPLC fractioning of the crude product mixture; Thermo Scientific ODS Hypersil column (250 × 4.6 mm, 5 µm); flow rate = 1.0 mL min^{-1}; linear gradient (10 to 40% over 25 min) of MeCN in 100 mM aq triethylammonium acetate (pH 7.0). The collected fractions are labeled E1–E5.

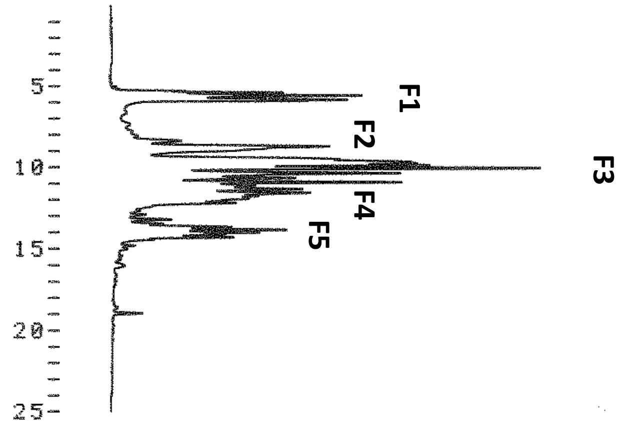

Figure S7: RP HPLC trace of fraction F of the IE HPLC fractioning of the crude product mixture; Thermo Scientific ODS Hypersil column (250 × 4.6 mm, 5 µm); flow rate = 1.0 mL min^{-1}; linear gradient (10 to 40% over 25 min) of MeCN in 100 mM aq triethylammonium acetate (pH 7.0). The collected fractions are labeled F1–F5.

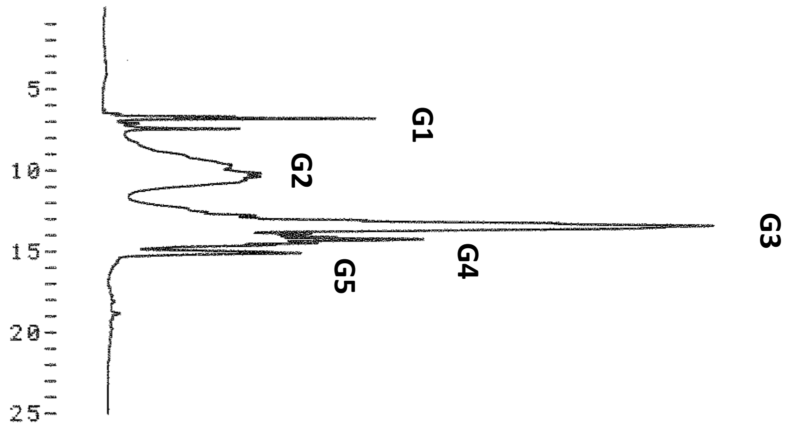

Figure S8: RP HPLC trace of fraction G of the IE HPLC fractioning of the crude product mixture; Thermo Scientific ODS Hypersil column (250 × 4.6 mm, 5 µm); flow rate = 1.0 mL min^{-1}; linear gradient (10 to 40% over 25 min) of MeCN in 100 mM aq triethylammonium acetate (pH 7.0). The collected fractions are labeled G1–G5.

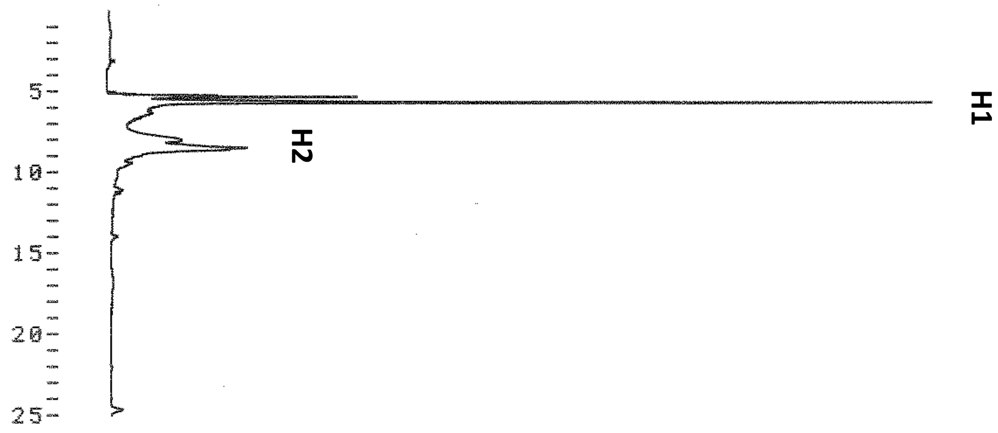

Figure S9: RP HPLC trace of fraction H of the IE HPLC fractioning of the crude product mixture; Thermo Scientific ODS Hypersil column (250 × 4.6 mm, 5 µm); flow rate = 1.0 mL min^{-1}; linear gradient (10 to 40% over 25 min) of MeCN in 100 mM aq triethylammonium acetate (pH 7.0). The collected fractions are labeled H1 and H2.

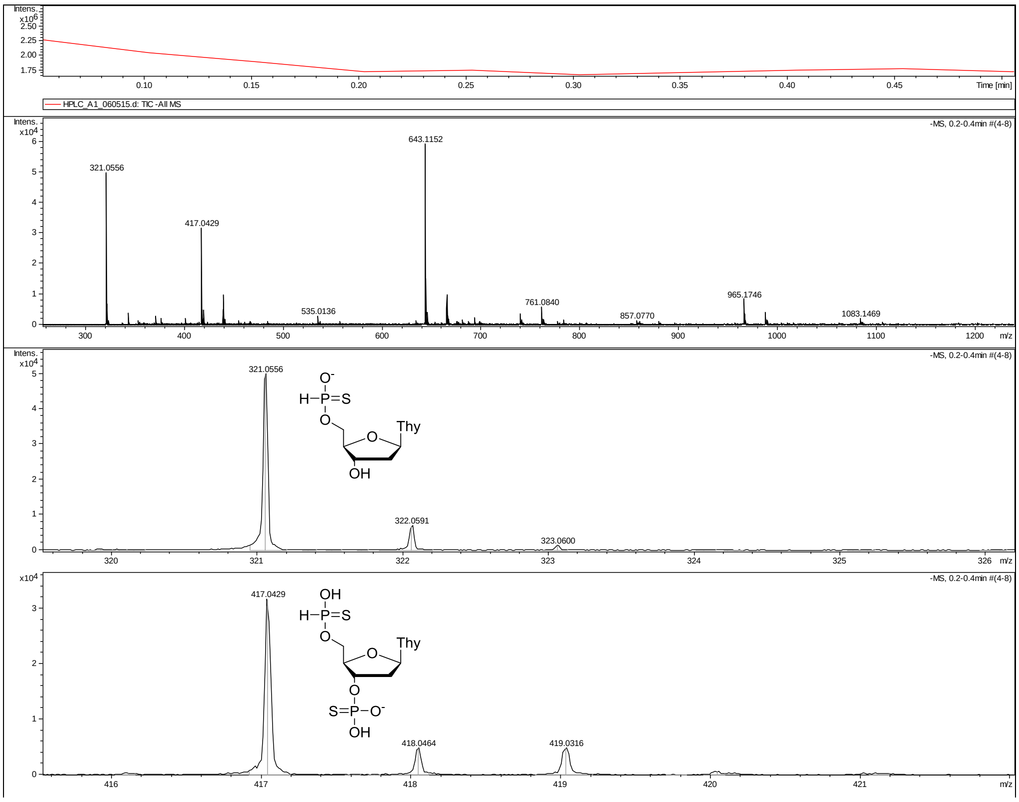

Figure S10: Electrospray ionization mass spectrum of fraction A1.

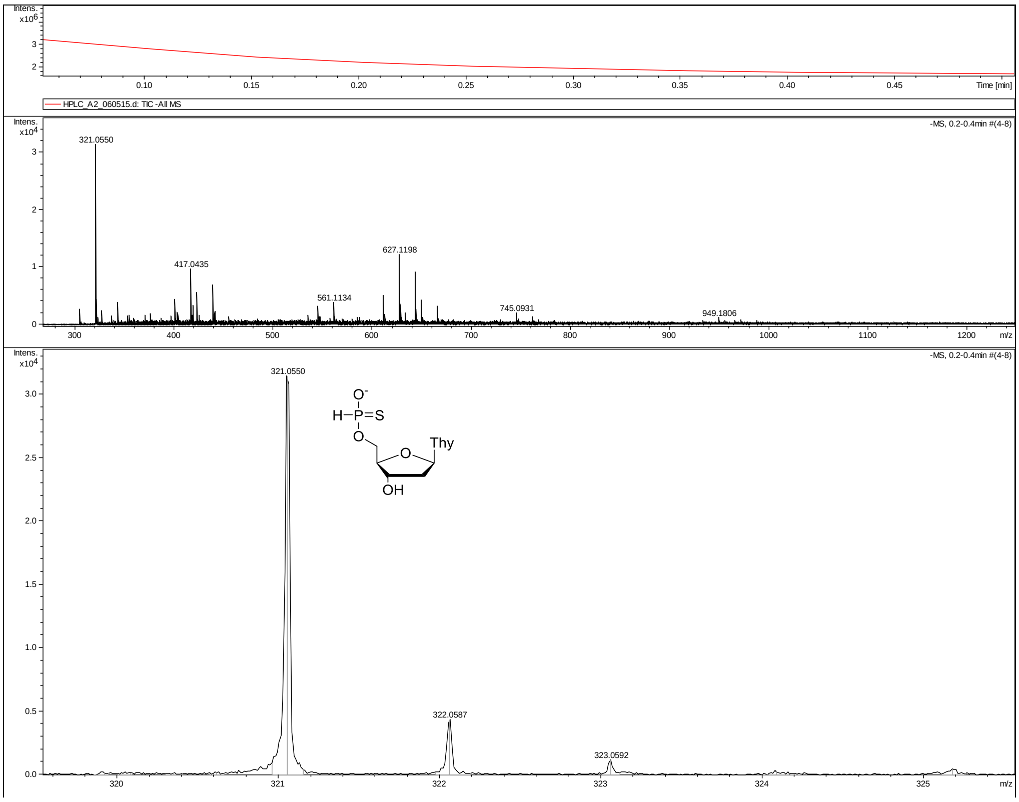

Figure S11: Electrospray ionization mass spectrum of fraction A2.

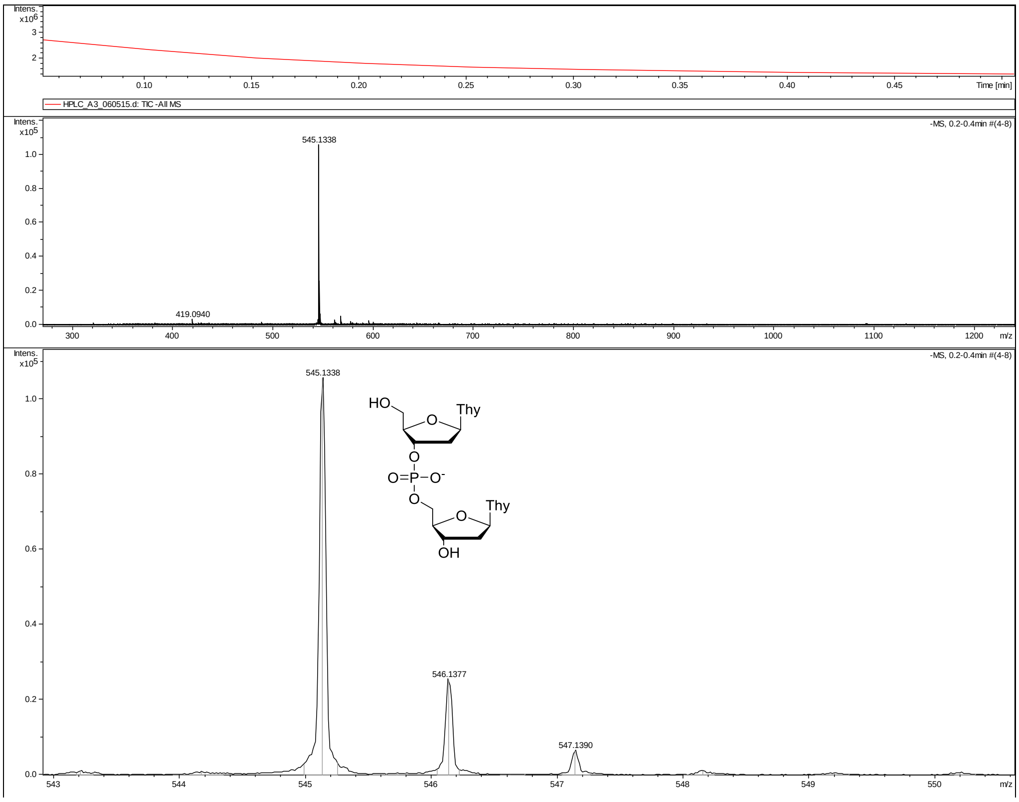

Figure S12: Electrospray ionization mass spectrum of fraction A3.

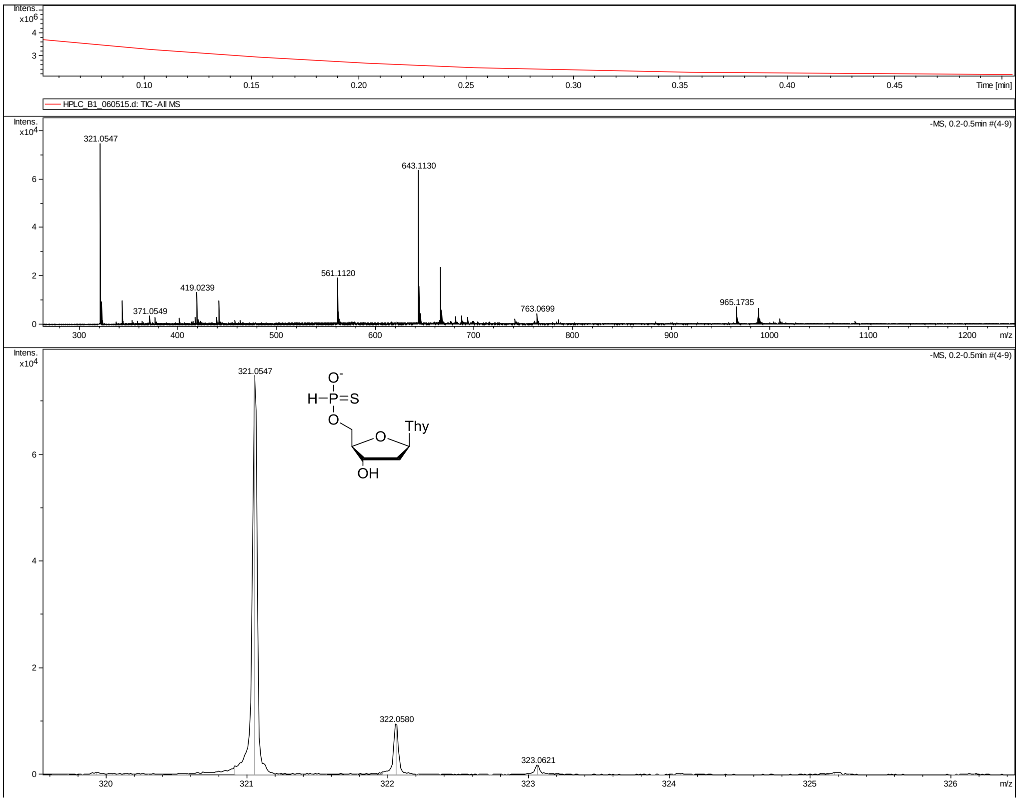

Figure S13: Electrospray ionization mass spectrum of fraction B1.

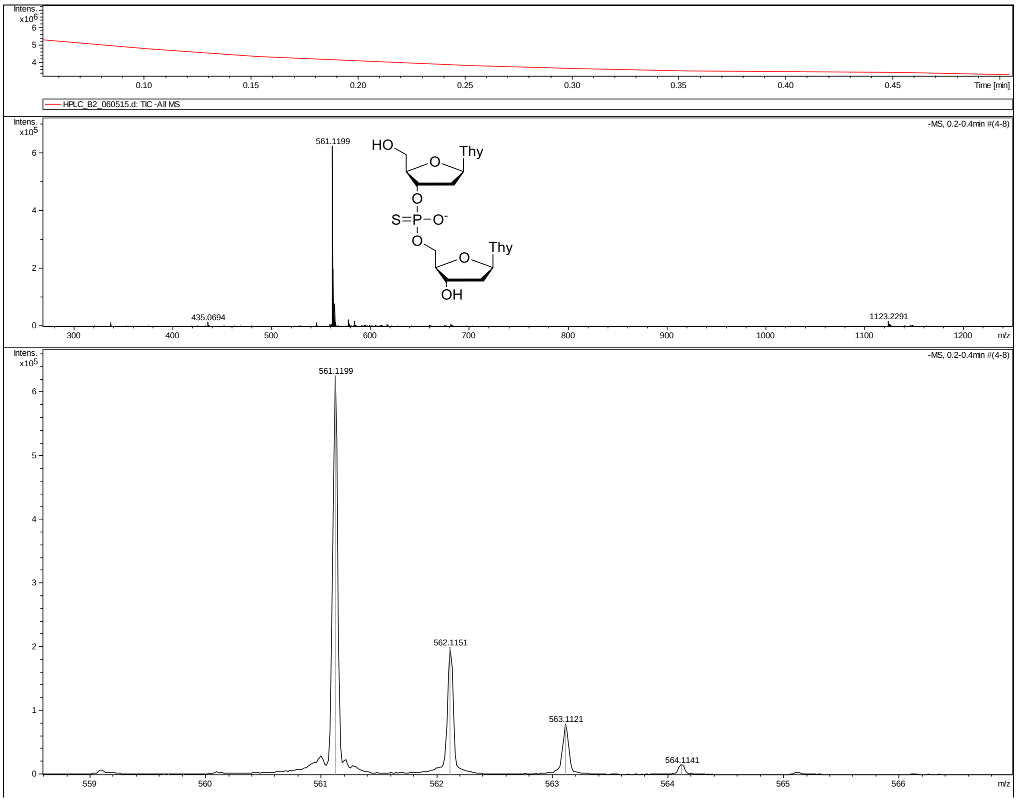

Figure S14: Electrospray ionization mass spectrum of fraction B2.

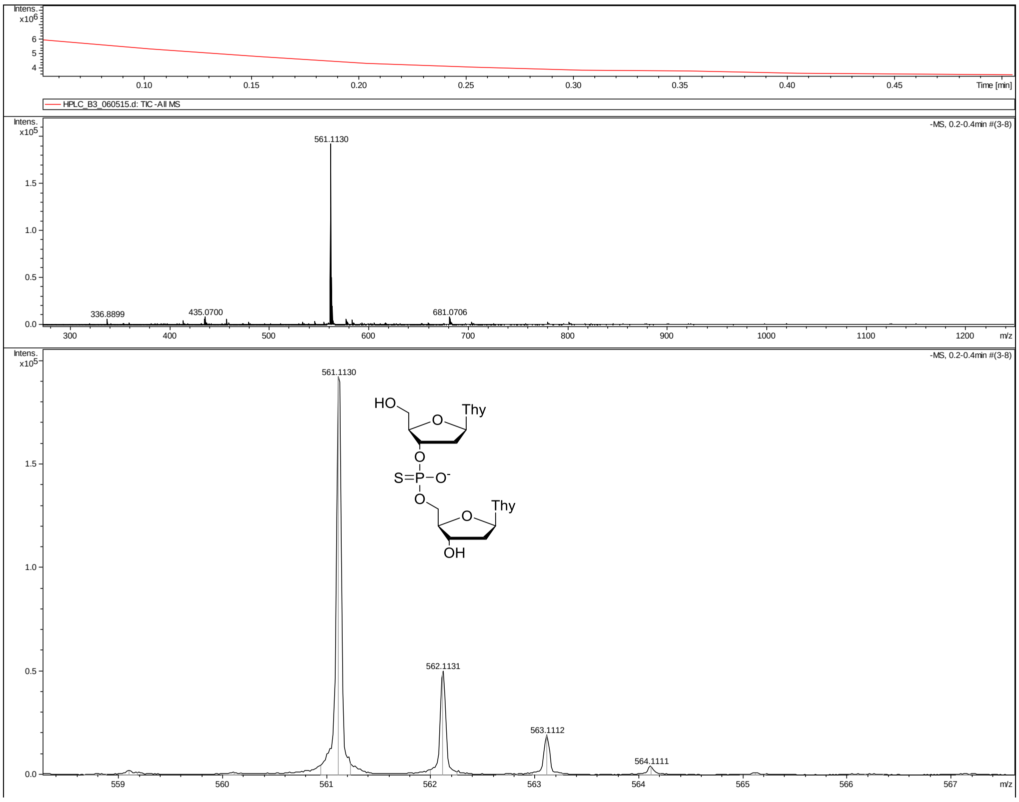

Figure S15: Electrospray ionization mass spectrum of fraction B3.

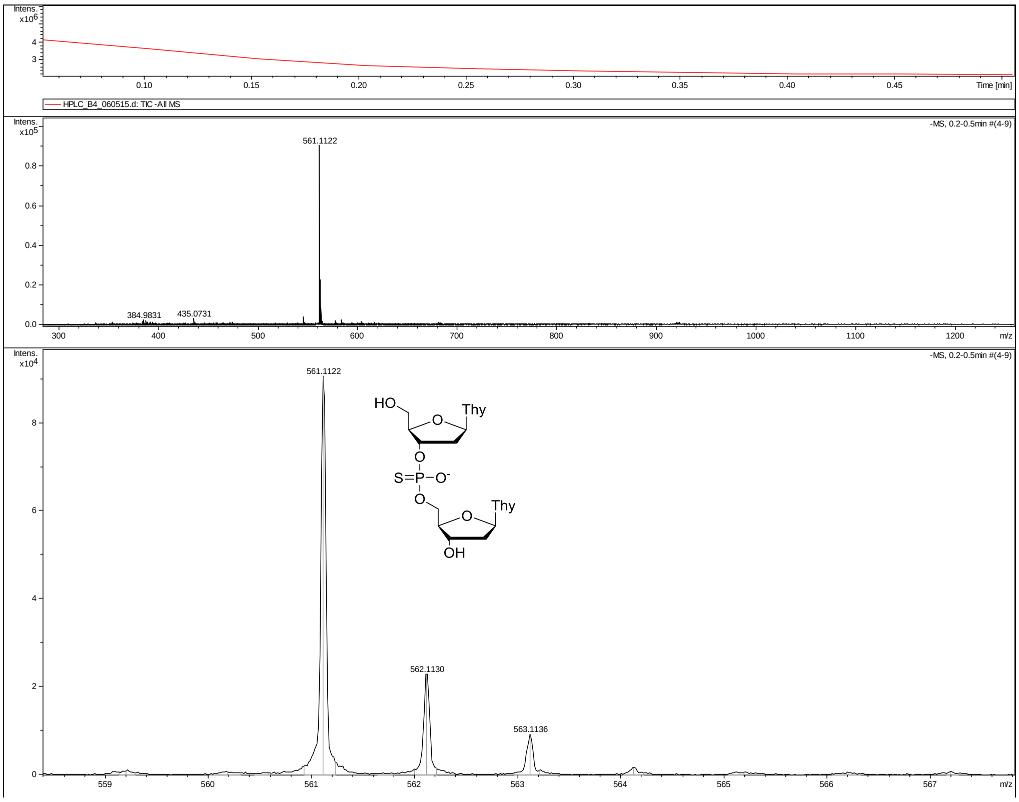

Figure S16: Electrospray ionization mass spectrum of fraction B4.

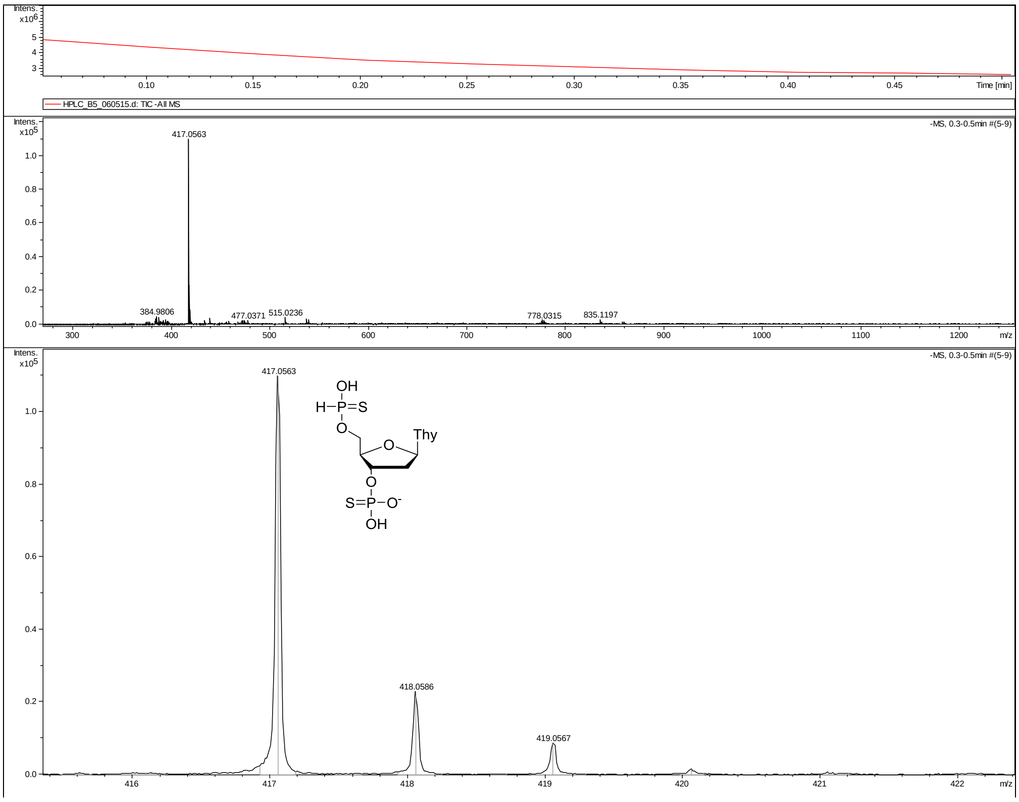

Figure S17: Electrospray ionization mass spectrum of fraction B5.

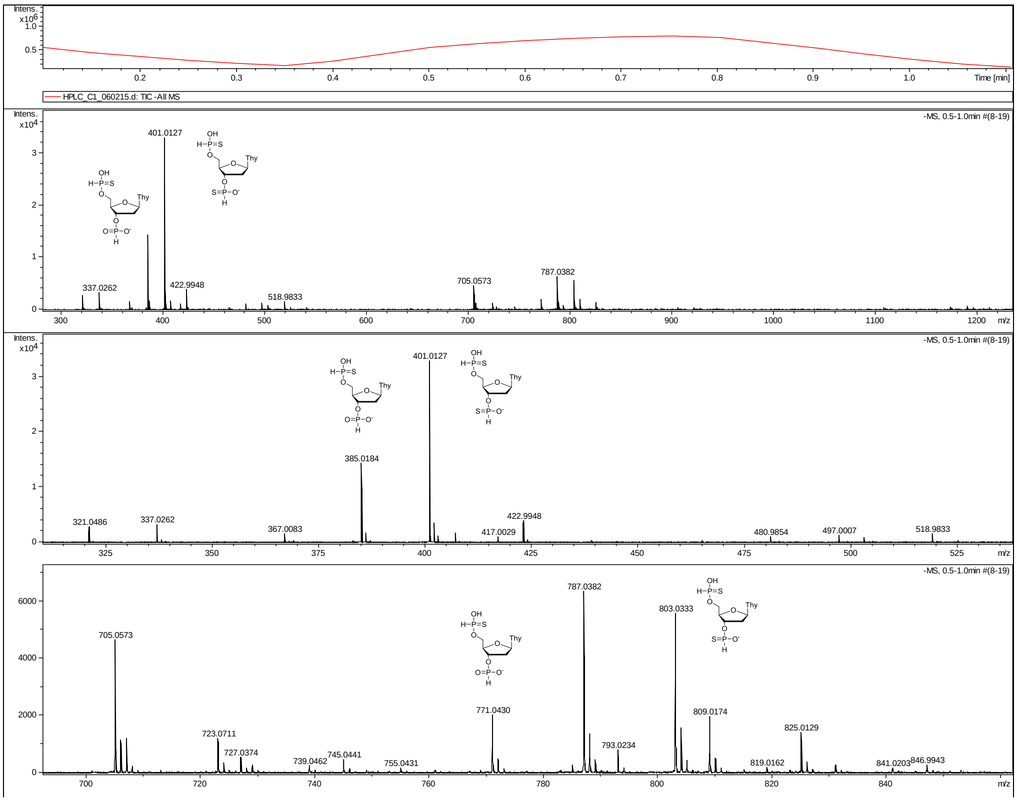

Figure S18: Electrospray ionization mass spectrum of fraction C1.

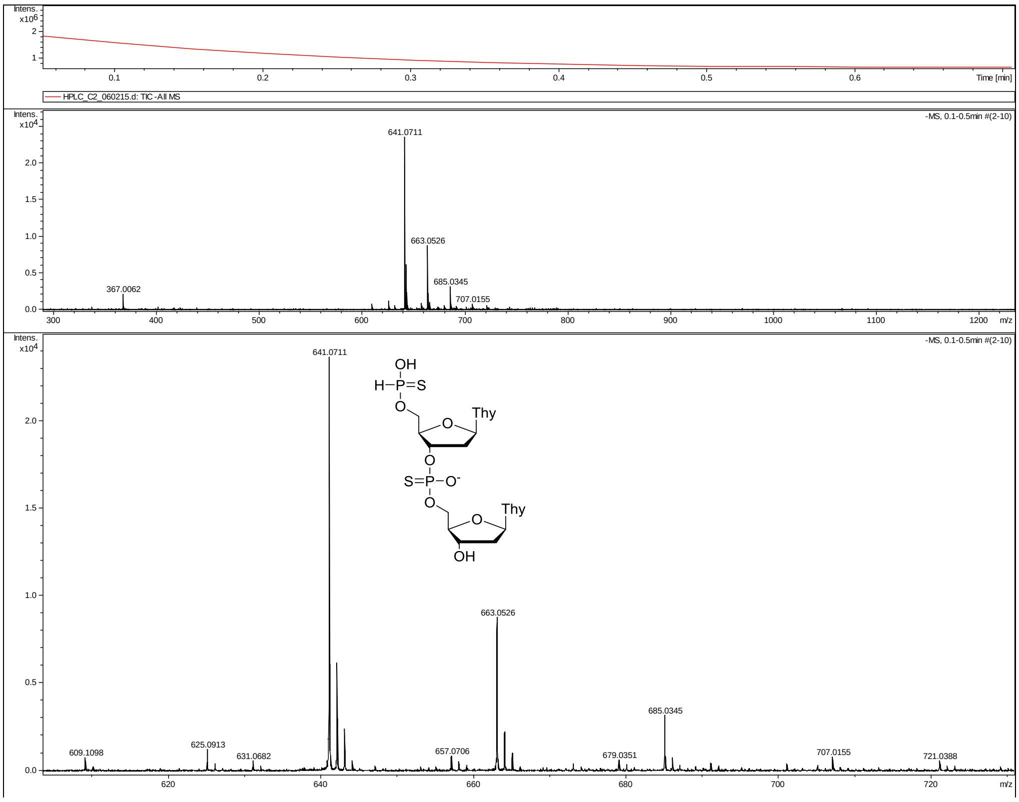

Figure S19: Electrospray ionization mass spectrum of fraction C2.

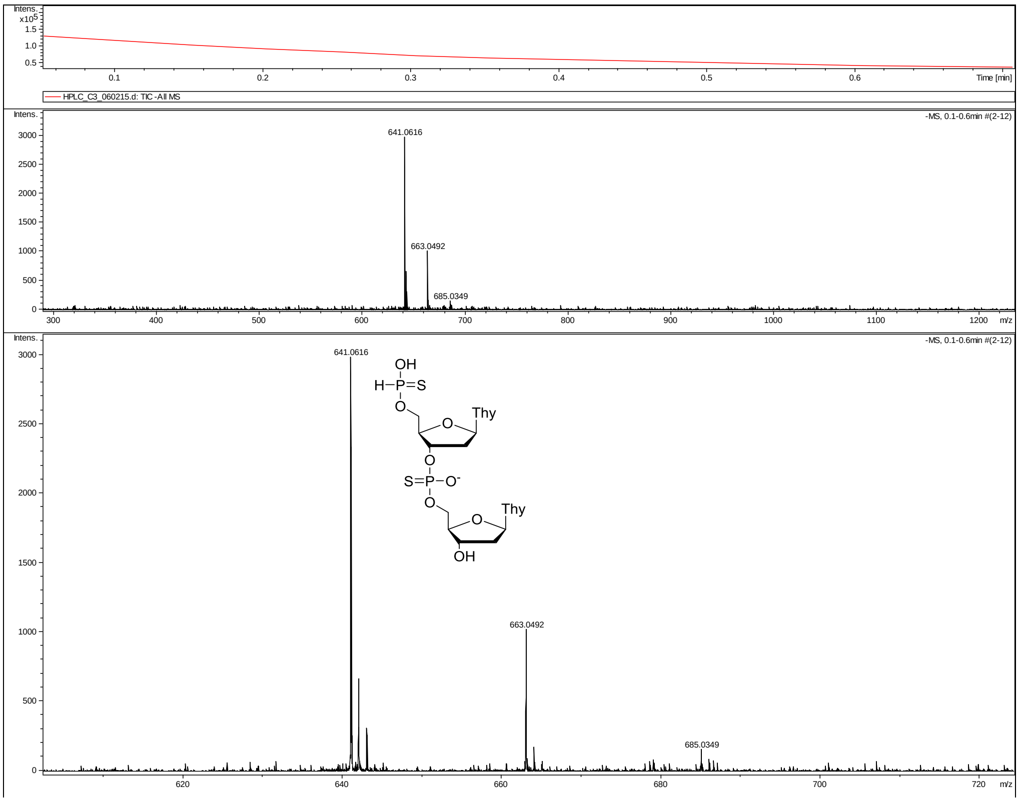

Figure S20: Electrospray ionization mass spectrum of fraction C3.

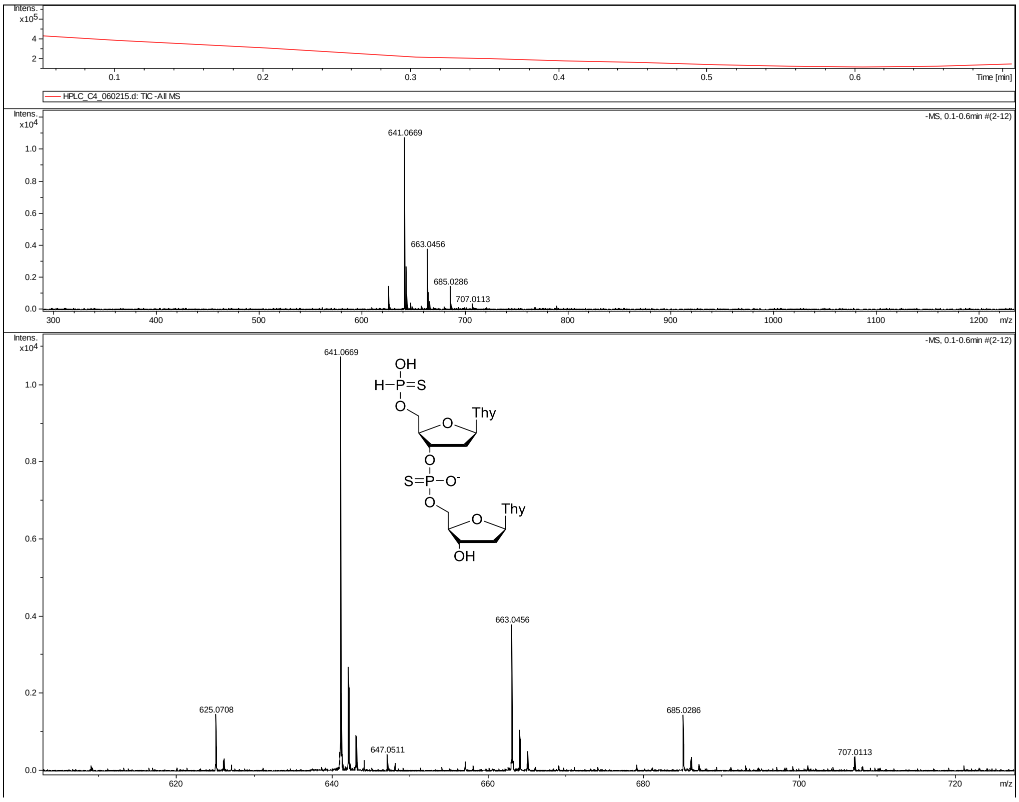

Figure S21: Electrospray ionization mass spectrum of fraction C4.

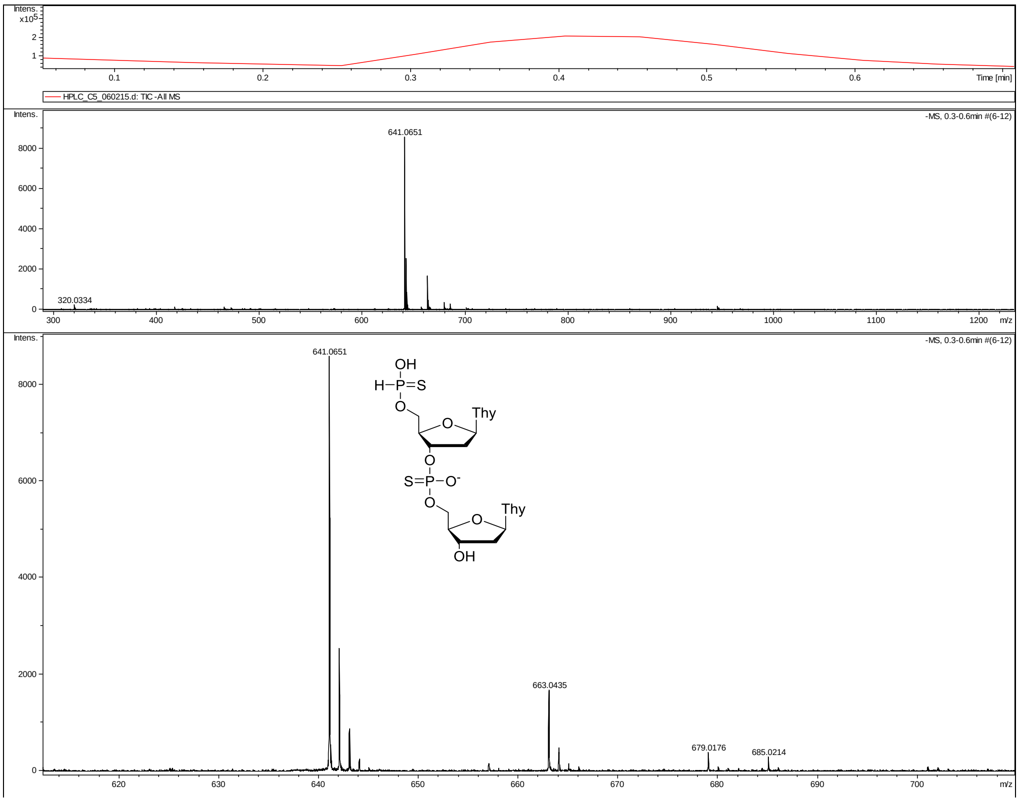

Figure S22: Electrospray ionization mass spectrum of fraction C5.

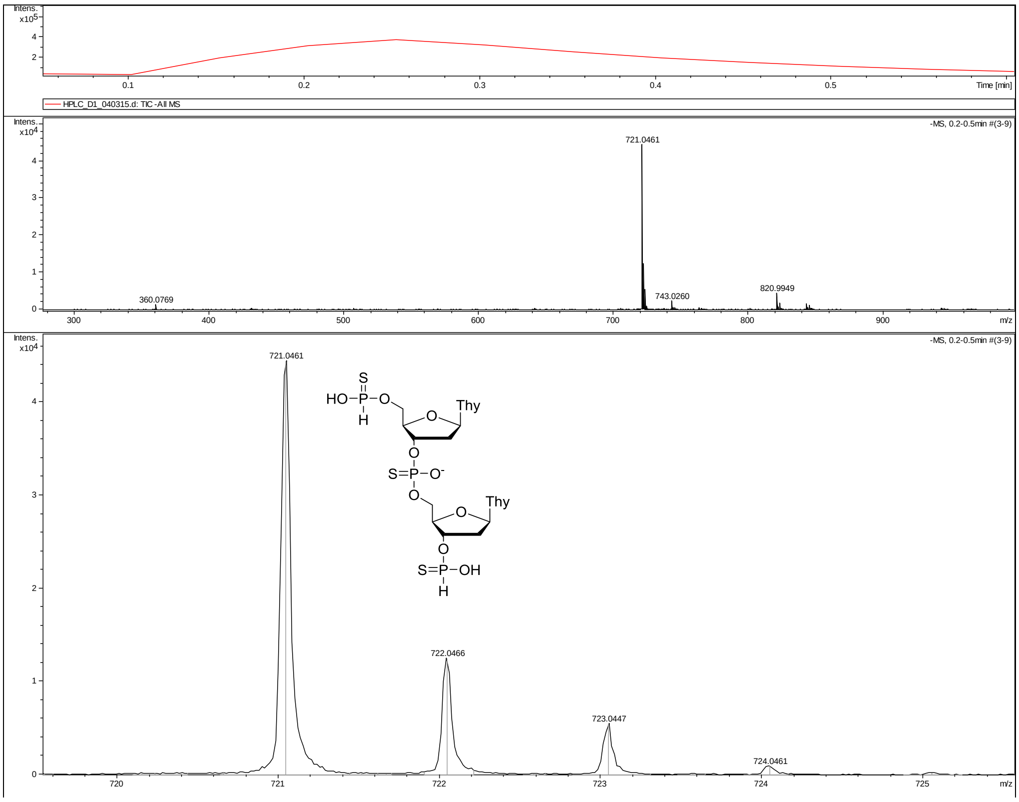

Figure S23: Electrospray ionization mass spectrum of fraction D1.

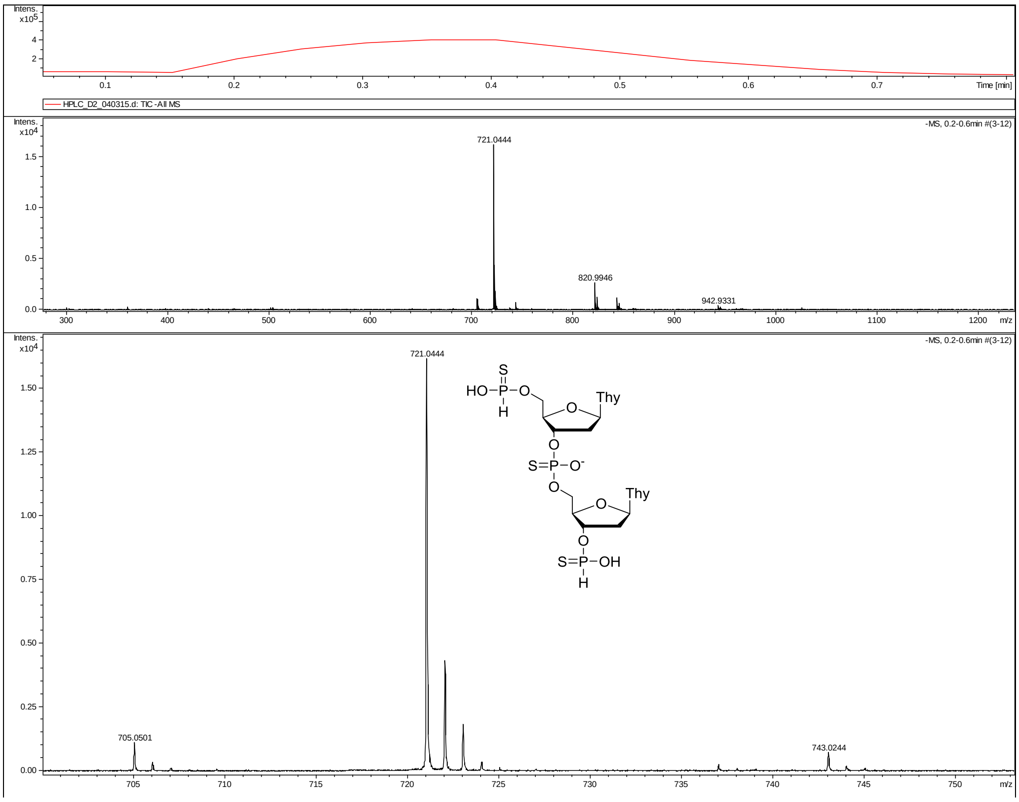

Figure S24: Electrospray ionization mass spectrum of fraction D2.

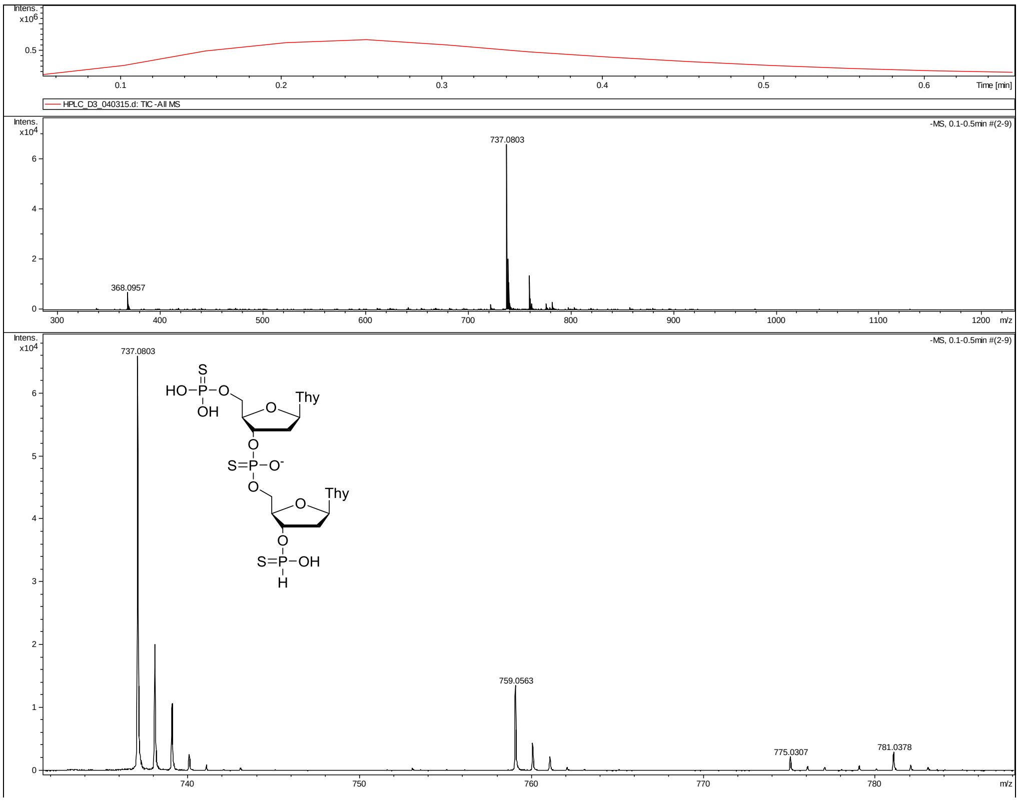

Figure S25: Electrospray ionization mass spectrum of fraction D3.

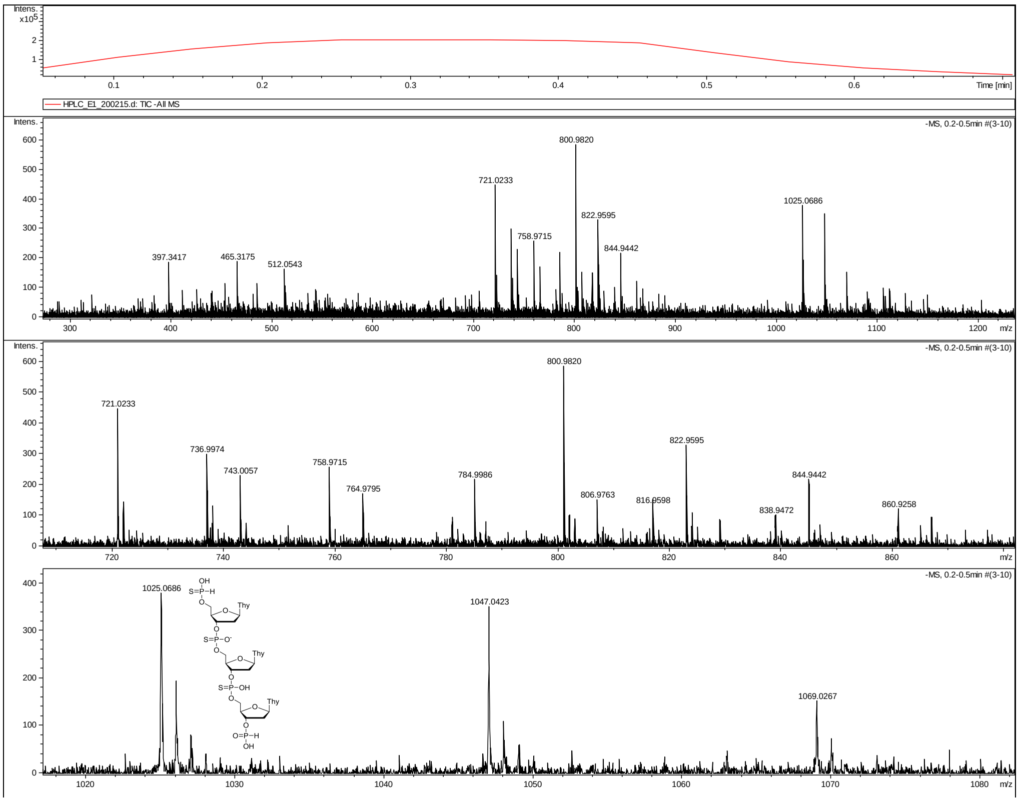

Figure S26: Electrospray ionization mass spectrum of fraction E1.

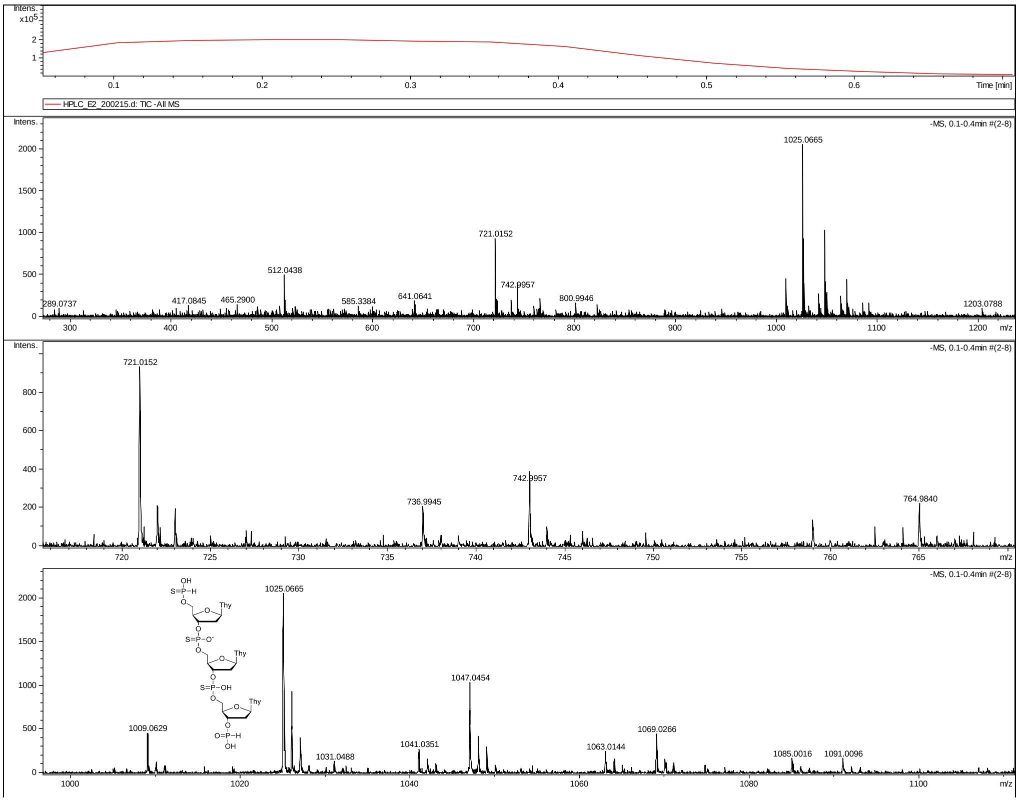

Figure S27: Electrospray ionization mass spectrum of fraction E2.

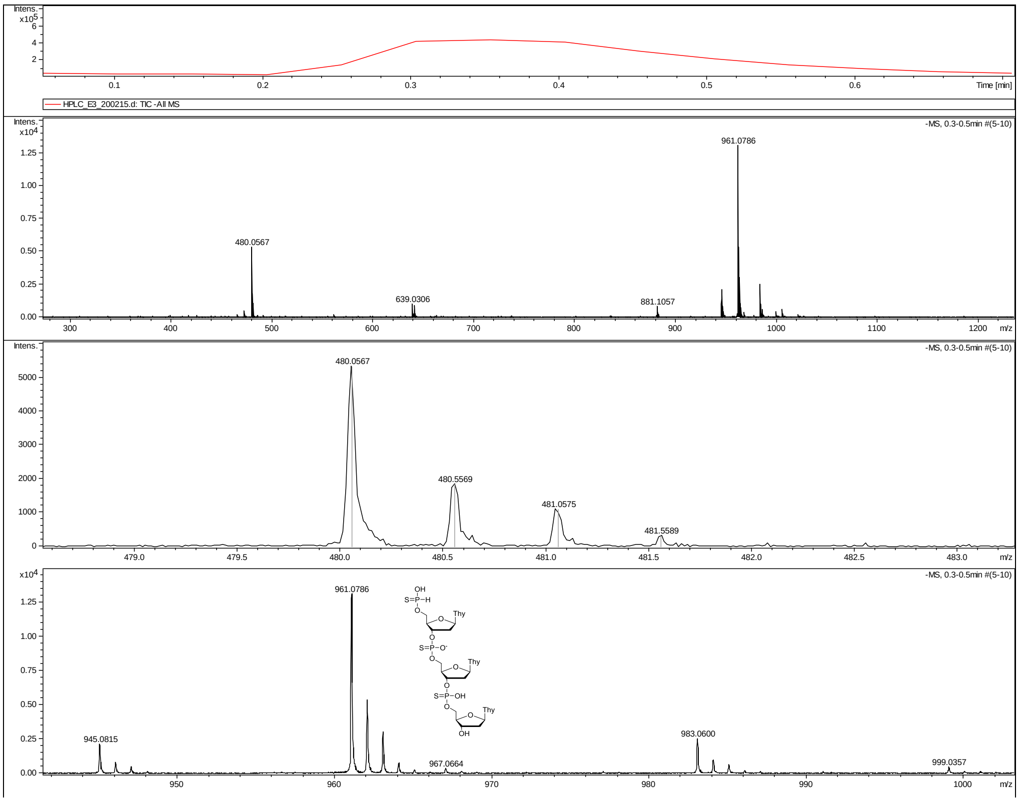

Figure S28: Electrospray ionization mass spectrum of fraction E3.

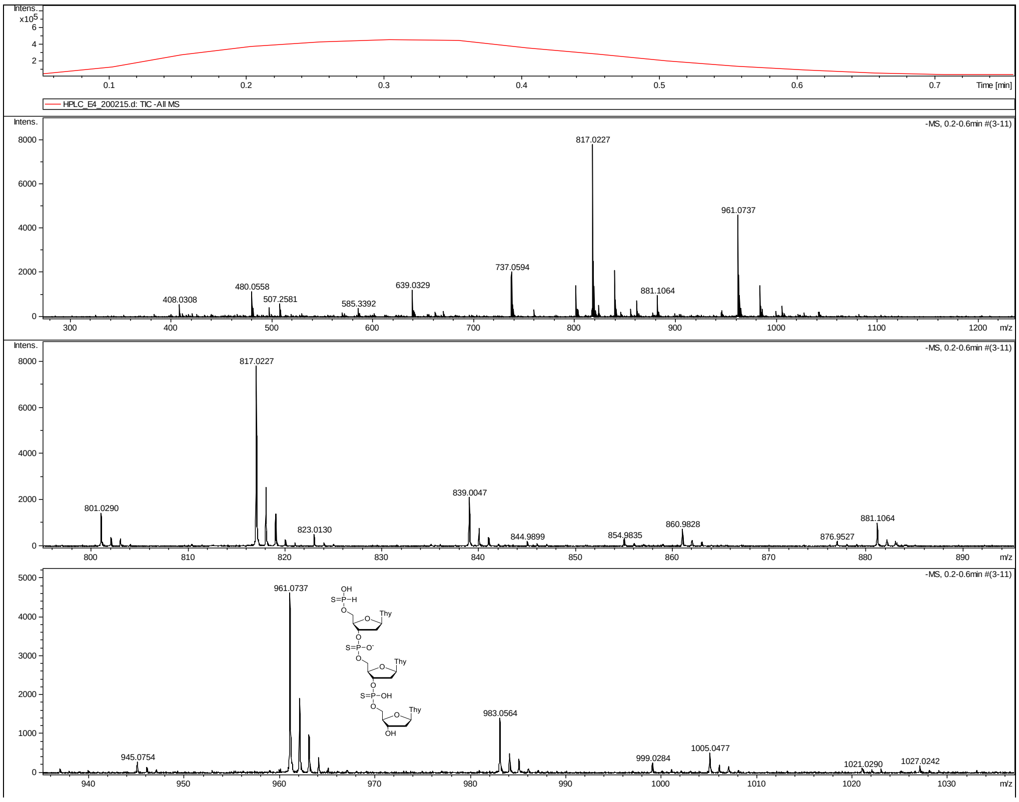

Figure S29: Electrospray ionization mass spectrum of fraction E4.

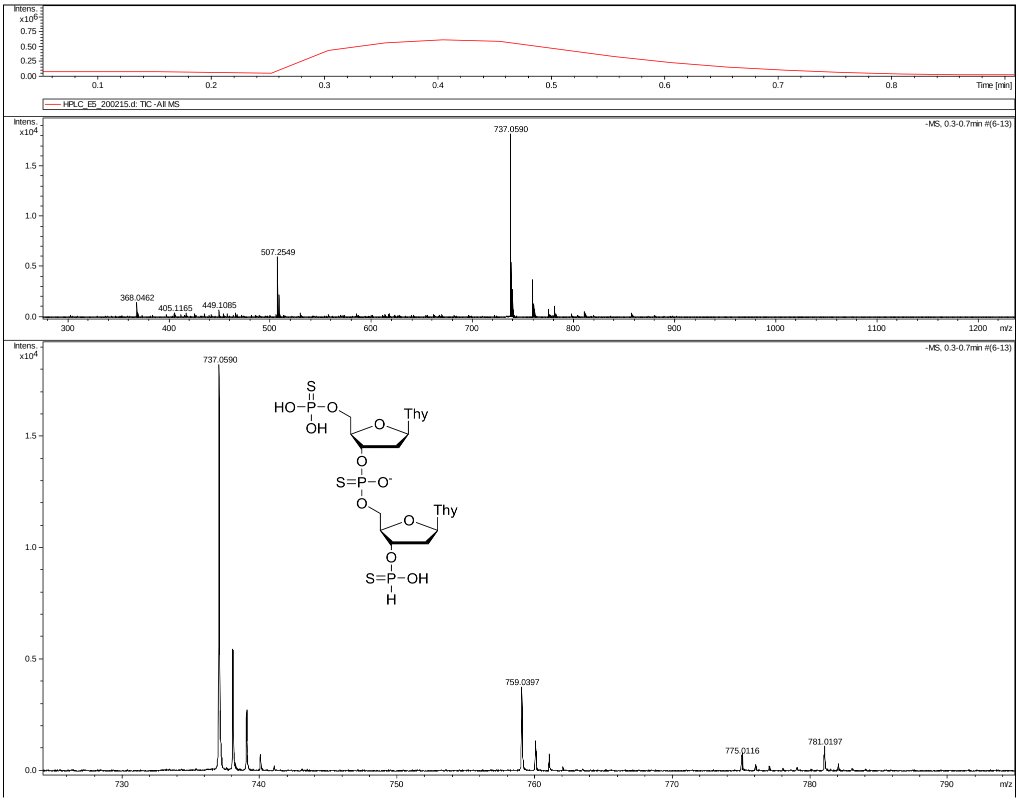

Figure S30: Electrospray ionization mass spectrum of fraction E5.

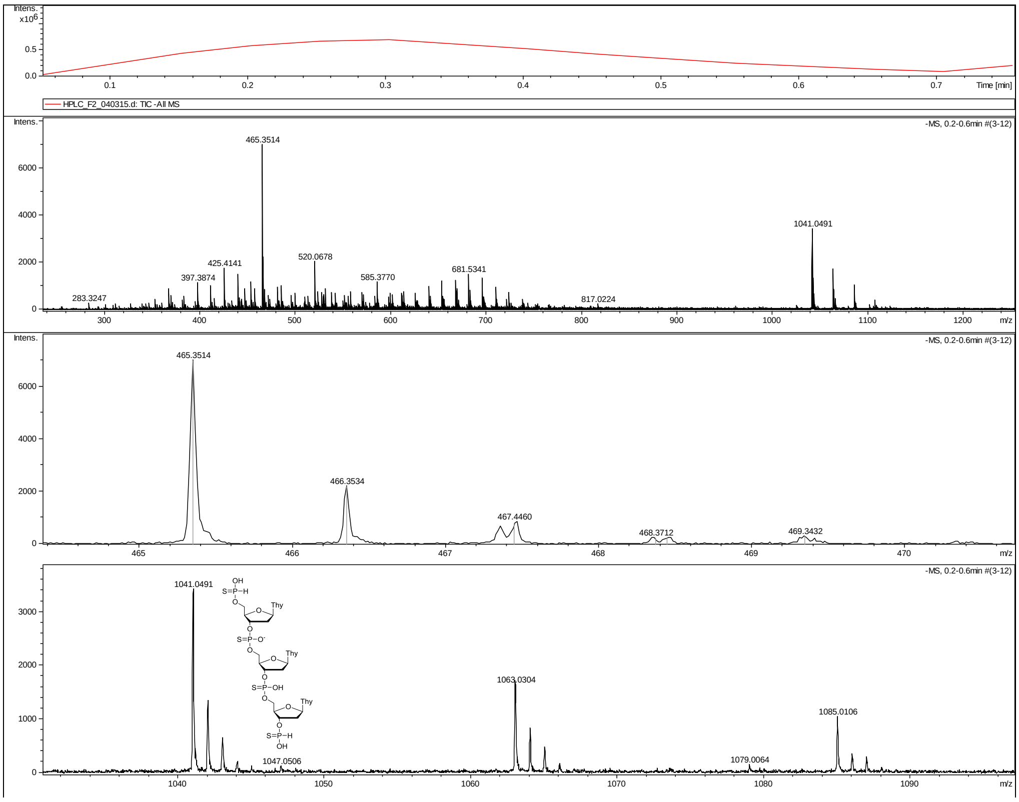

Figure S31: Electrospray ionization mass spectrum of fraction F2.

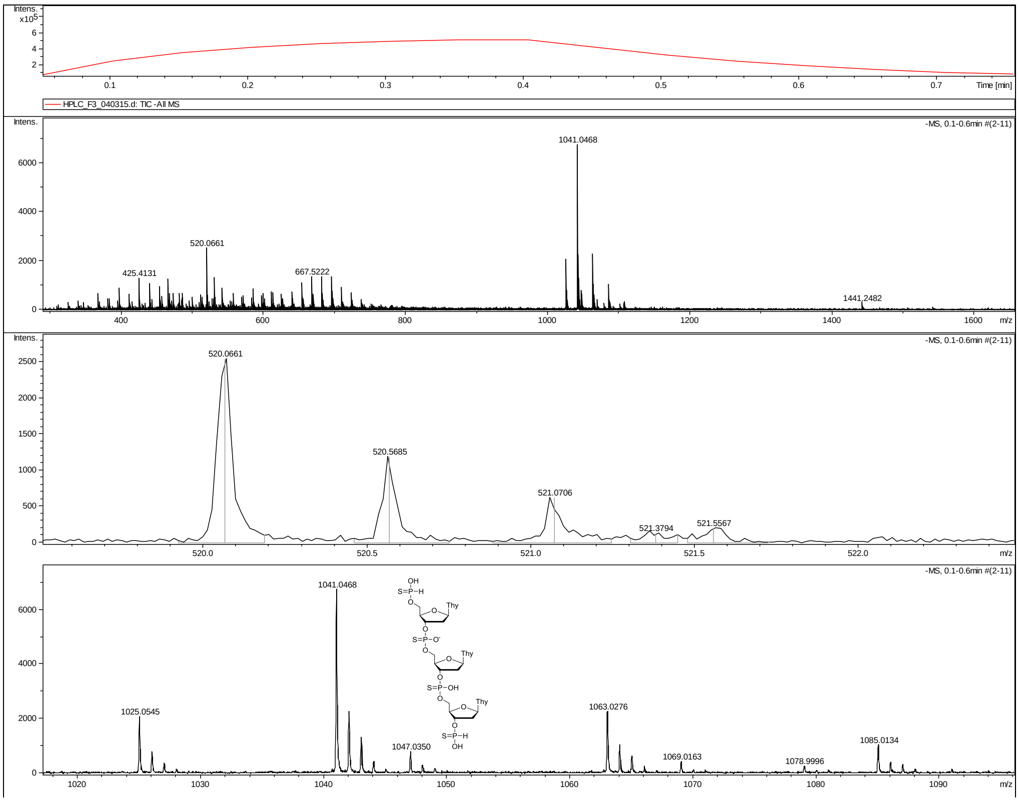

Figure S32: Electrospray ionization mass spectrum of fraction F3.

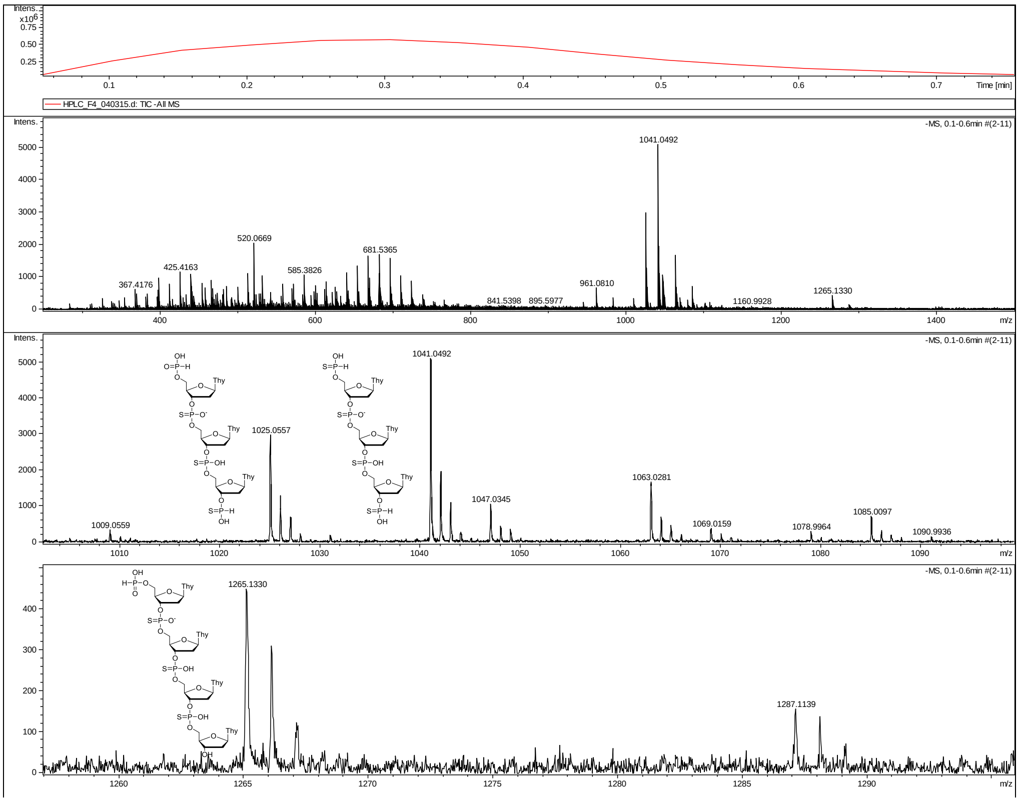

Figure S33: Electrospray ionization mass spectrum of fraction F4.

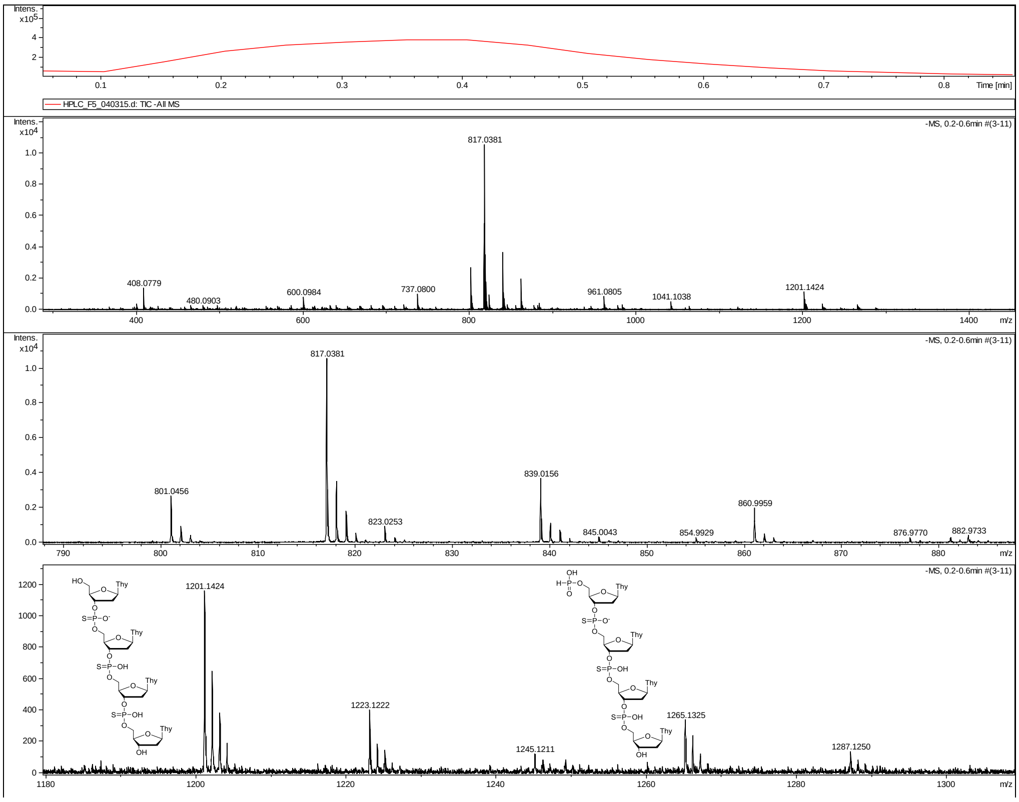

Figure S34: Electrospray ionization mass spectrum of fraction F5.

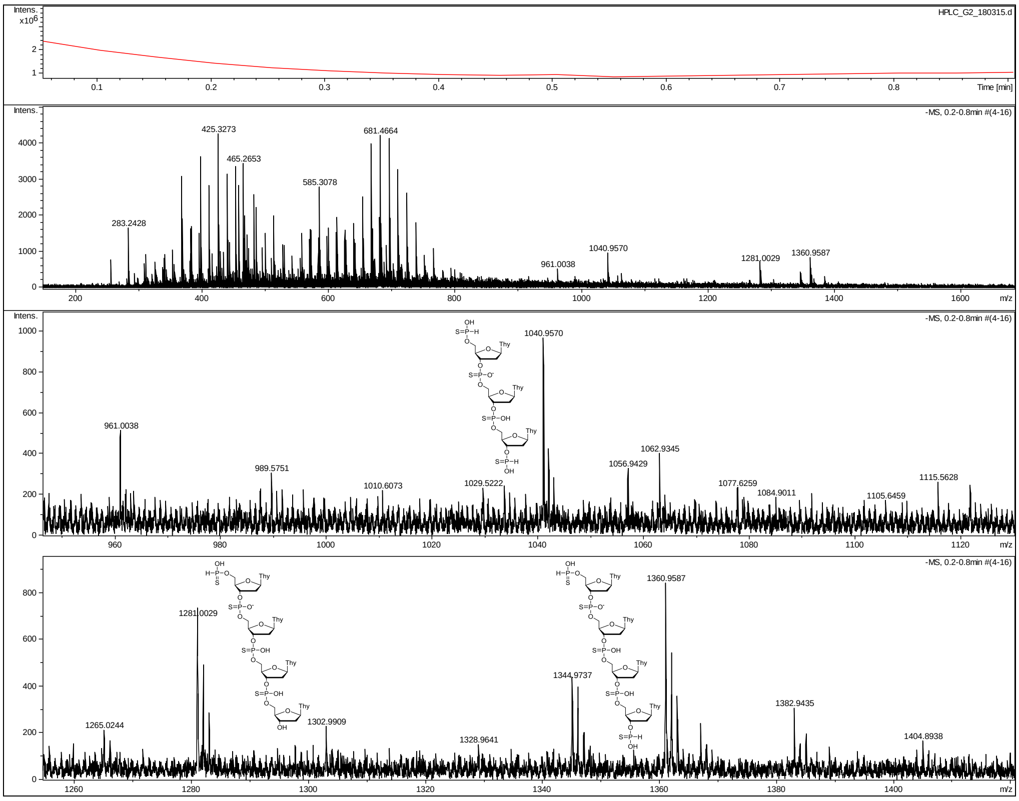

Figure S35: Electrospray ionization mass spectrum of fraction G2.

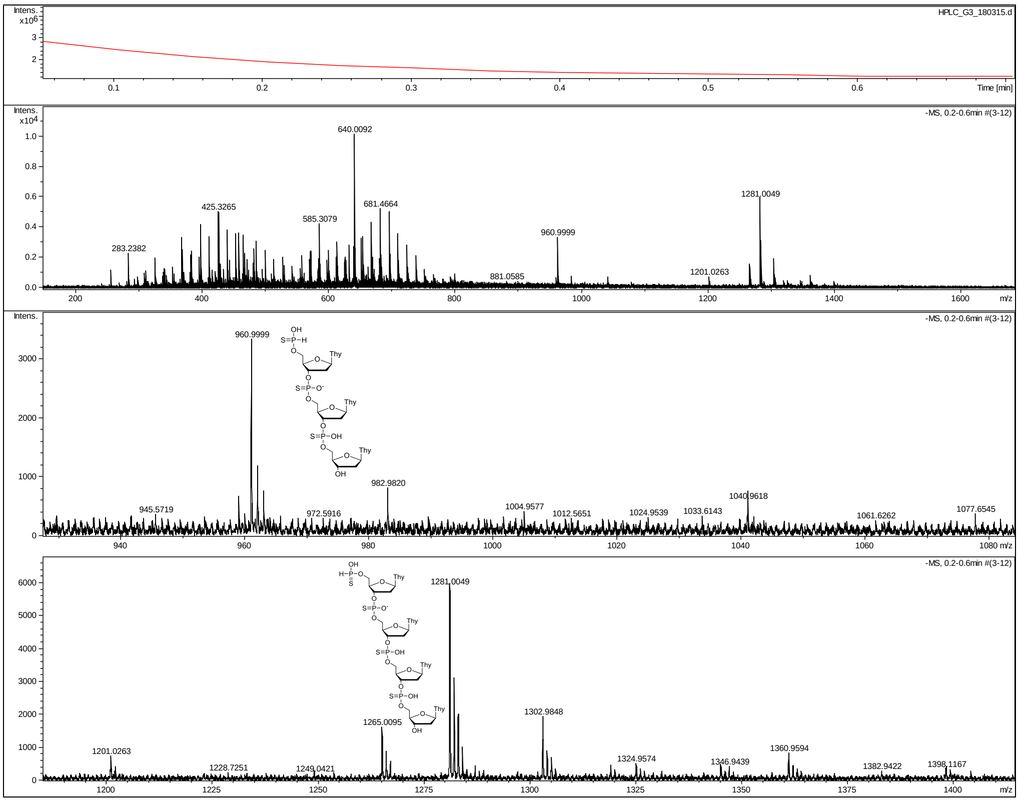

Figure S36: Electrospray ionization mass spectrum of fraction G3.

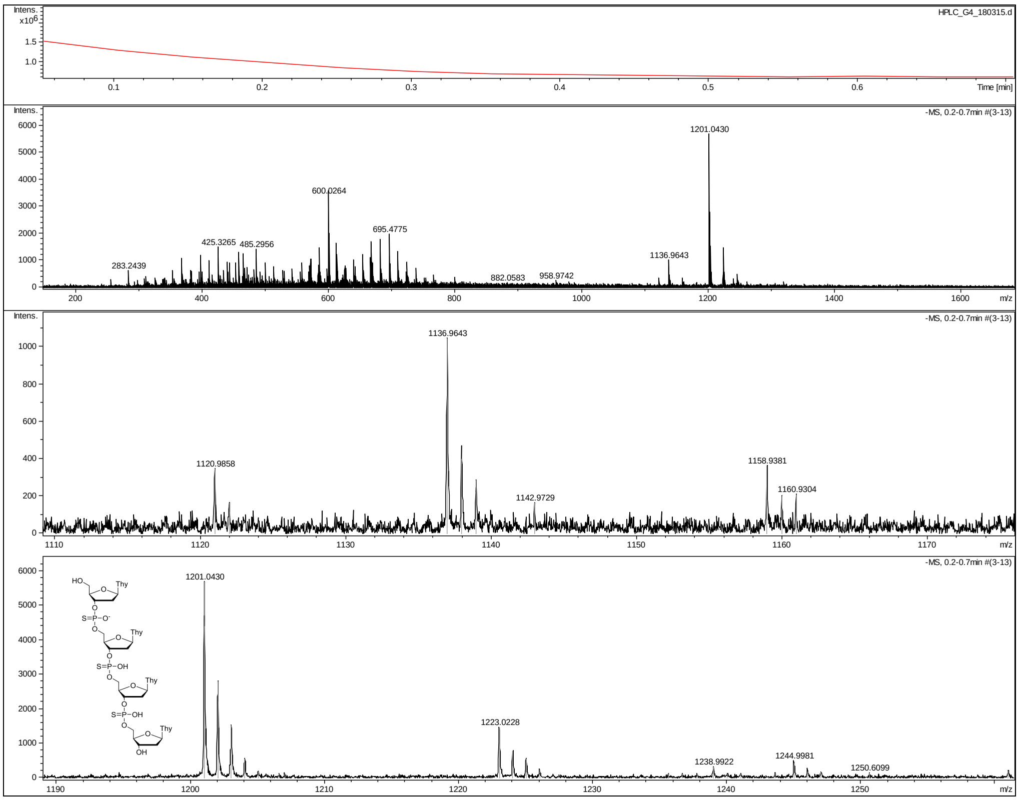

Figure S37: Electrospray ionization mass spectrum of fraction G4.

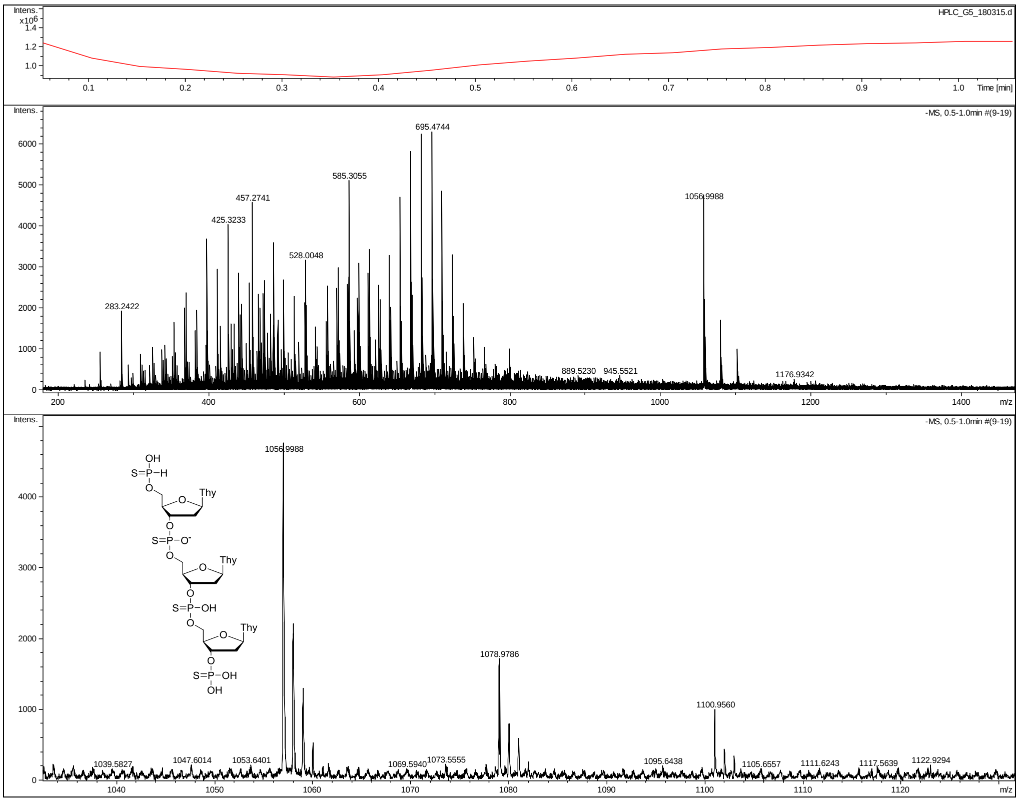

Figure S38: Electrospray ionization mass spectrum of fraction G5.

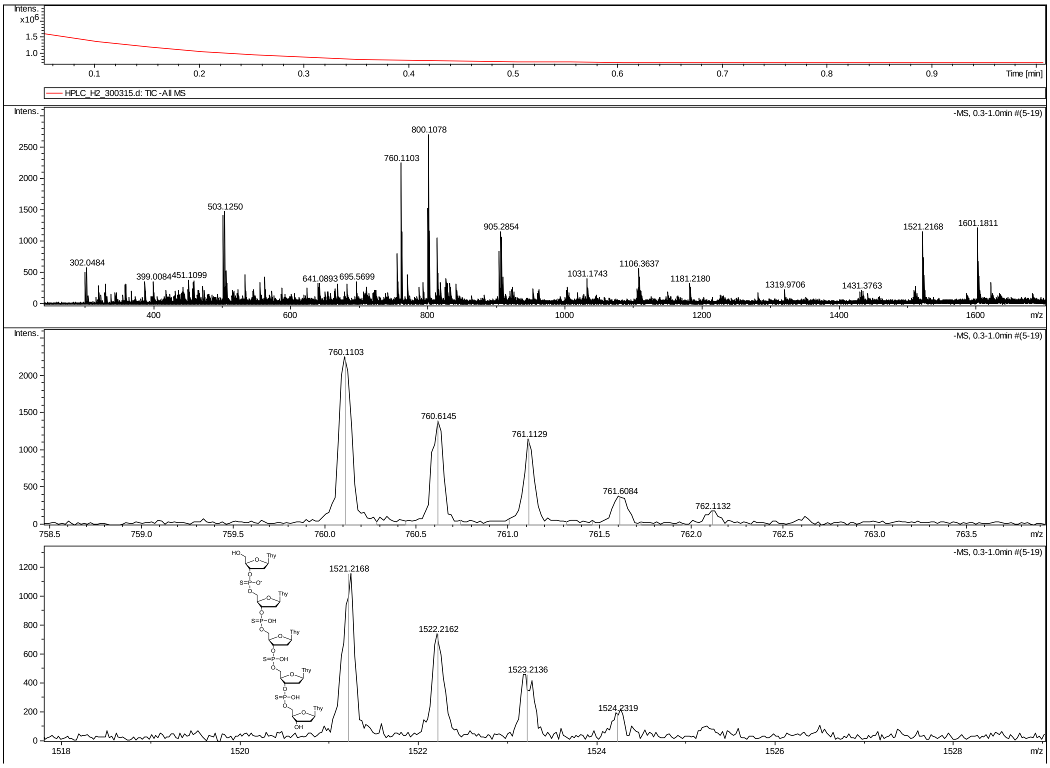

Figure S39: Electrospray ionization mass spectrum of fraction H2.

Desulfurization and enzymatic digestion of the phosphorothioate oligonucleotide products

To a sample of a product fraction consisting mainly of tetrameric phosphorothioate oligonucleotides (G3, Figure S36, approximately 0.25 nmol in 2.5 µL of water), a 26.3 mmol L^{-1} solution of iodine in pyridine (7.5 µL) was added. The resulting mixture was incubated at 37 °C for 2 h, after which the reaction was quenched by addition of 10 mmol L^{-1} aq $NaHSO_3$ (20 µL). The product mixture was diluted with water (1.0 mL) and lyophilized. The residue was dissolved in 25 mmol L^{-1} TRIS buffer (pH 7.0) and a solution of P1 nuclease (approximately 10 µg in 1.0 µL of water) was added. The solution was incubated at 37 °C for 2 h, after which it was analyzed by anion-exchange HPLC on a Dionex DNASwift™ column (150 × 5 mm, monolithic) eluting with a linear gradient (2 to 35% over 20 min) of 330 mM $NaClO_4$ in 20 mM TRIS buffer (pH 7.0). The flow rate was 1.5 mL min^{-1} and the detection wavelength 275 nm. Approximately 78% of the oligonucleotide material had reacted to monomeric products (thymidine and thymidine-5´-monophosphate).
